# An emotional discrete controller PSO tuned and designed for a real industrial pumping system

**DOI:** 10.1038/s41598-022-08192-2

**Published:** 2022-03-11

**Authors:** Davidson C. Marques, Jeydson L. Silva, Milde Maria S. Lira, Ronaldo R. B. Aquino

**Affiliations:** Federal University of Pernambuco, DEE, Recife, 50740-530 Brazil

**Keywords:** Electrical and electronic engineering, Computational platforms and environments

## Abstract

The application of automation techniques to water pump systems, combined with modern control techniques, has been increasing the hydraulic and energy efficiency of such systems. In this context, the objective of this work is to present an intelligent method of flow control based on Brain Emotional Learning Basic Intelligent Control (BELBIC), which will be applied to an experimental workbench of a pumping system, located in the Energy Efficiency and Energy Quality Laboratory (LEEQE) at Federal University of Pernambuco (UFPE). The parameters of this controller are optimized with a particle swarm optimization (PSO) technique with minimization of Integral Absolute Error (IAE). Initial tests were performed in a computational environment so that the system’s performance could be pre-tested, thereby the dynamics of the system was modeled from real data generated in the process. The experimental results were obtained through the implementation of this control system in a programmable logic controller (PLC), which was the device responsible for all the automation of the workbench previously mentioned. The data of this workbench were collected using a supervisory system exclusively developed for this work. These data were then used to analyze the performance of the proposed control system, which demonstrated that its behavior was efficient.

## Introduction

The sanitation sector has a high rate of technical losses of water captation along its final distribution. This statement is related to the fact that many water distribution plants have: choke valves; pressure losses in piping; system oversizing and obsolescence of motors; low efficiency motors; besides low investments towards automation and micrometering. Due to these facts, companies have aimed at investing in technologies to minimize these losses, targeting a potential huge market for applications in supervision and control system; load curve modulation; equipment replacement and applications using frequency converters.

When using throttling valves to control the flow, maneuvers are performed according to operational demands. On the pump extremity, a valve is inserted with the purpose of altering the system flow rate by reducing the diameter, which increases its resistance. The load torque registered by the motor decreases as the power decreases, and the speed is kept almost constant. The practice of this operation increases local loss of pressure, and raises the upstream of the load control valve^1^. Therefore, the lifespan of the equipment will be impaired and the excess of energy may cause vibrations, damaging the pipes, the pumping systems and valves.

Therefore, sanitation companies are slowly and selectively implementing the strategy of automation of water supply systems in order to reduce the chance of improper maneuvering and improper operation by operators, to optimize manpower, in addition to leakage control, cost reduction in electricity consumption and equipment maintenance.

Although scientific research in this area is of utmost importance, it is still rare, as it presents difficulties in the dynamics of these systems, usually nonlinear and time-varying. Most controllers used are based on “conventional” PID-type control techniques developed and coupled by equipment manufacturers^2^.

The aim of this work is not to confront other controls, but to show the application of BELBIC as an option in industrial systems. And considering that PID controllers are widespread in the industrial automation area that use Programmable Logic Controllers. This type of control is easy to implement, as they are native to most PLC’s. Therefore, the work brings an innovative alternative in the implementation of a controller of this size in a PLC, which does not provide any other control besides the PID. So the work concludes that it will be possible to have a new alternative for linear or non-linear systems when it comes to applications involving PLC.

In^3^, the authors presented a pressure control methodology in supply systems using a Programmable Logic Controller (PLC) and a frequency inverter, with the PLC being responsible for the control logic and the frequency inverter for pressure regulation. Through the logic developed, and the PID controller of the PLC, adjusted accordingly to the process, it was possible to control the motor drive’s frequency and the automatic definition of the amount of pumps in operation, controlling the flow and maintaining constant pressure.

As smart control techniques used in artificial intelligence associated with automation may have increased hydraulic and energy efficiency in most water supply systems, since they can have real time monitoring and control of the system’s various sectors, reducing significantly the operating costs and quality in the supply.

Artificial intelligence has awakened growing interest in the control and automation community by exploring intelligent control techniques based on artificial neural networks^4^ and fuzzy logic^5^ to solve control and optimization problems.Despite the great advances involving artificial intelligence (AI), its vulnerability of artificial intelligence against disturbances in general, can significantly restrict its applicability in critical security systems. On the other hand, fuzzy systems have some limitations, mainly due to their approach completely dependent on human knowledge and experience^6^. However, a computational model emotional learning in the amygdala was introduced by two researchers, who computationally modeled the emotional learning of the mammalian brain^7,8^, and then called it Brain Emotional Learning Based Intelligent Controller (BELBIC). It reproduces the regions of the amygdala, orbitofrontal cortex (OFC), thalamus, and sensory input cortex, that are usually responsible for processing the brain’s emotions.

BELBIC is a technique with relatively low computational load complexity, which makes it viable for practical applications in real time. This system has a reinforcement learning approach as a principle, being effective to deal with uncertainties and disturbances in the system. However, unlike the reinforcement learning techniques in the machine learning area, BELBIC does not present the need for exhaustive training.

Some studies point that the BELBIC controller has been successfully used to make decisions and to control simple linear systems as in^9^, and also in nonlinear systems such as the control of a command system of a magnetic synchronous motor and automatic voltage regulator (AVR)^10,11^, level control of quadruple tank system^12^, Multi-variable Adaptive Stimuli for an Emotional Learning Based Controller for a MIMO Process^13^, Development of a Hybrid Path Planning Algorithm and a Bio-Inspired Control for an Omni-Wheel Mobile Robot^14^, micro heat exchanger^15^, flight control^16^ and crane positioning and displacement control^17^, control method applied in an industrial fan system compared to the conventional PI controller^18^, control industrial induction heating systems with a serie of resonant inverters and compare performance with the conventional PID controller^19^, features an application for accurate tracking of the speed of the hybrid stepper motor^20^. On the other hand, new BELBIC models have been developed in^21^ and^22^. In^21^ a proposed G-BELBIC applies a nonlinear learning module with universal approximation (UA) property and can be applicable in various model based learning controller (MBC) engineering applications. And^22^ presents a Self-Organizing Brain Emotional Learning Controller of Mobile Robots. That’s why, the BELBIC has shown capable of controlling a diversity nonlinear dynamic system^23,24^.

In general, BELBIC has several advantages of use, such as quick response to changes in system dynamics, has an typical configuration, free model, high flexibility and the like, which is why it has been successfully used in several scientific researches.

This paper presents the implementation of the BELBIC controller in a Siemens SIMATIC S7-300 CPU 313C-2DP for flow control of the LEEQE industrial pumping system. The choice of controller was motivated by presenting peculiar characteristics in the selection of parameters, thus obtaining the possibility of achieving the most suitable response in nonlinear systems^25,26^. As the parameter adjustment has a flexibility, a method of optimization was proposed as an alternative to estimate the best BELBIC parameters with the objective minimization function of the IAE.

The stochastic optimization method used in this paper was the PSO, introduced in 1995 by Kennedy and Eberhart^27,28^. The PSO resembles other evolutionary computation techniques, such as Genetic Algorithms (GA)^29^. It is considered an algorithm easily implemented because it has few gains to be adjusted. Widely applied in several problems of function optimizations, fuzzy controllers and feed-forward neural networks training (FFNN)^30^.

Initially, the system was simulated in a computational environment for the dynamics analysis of its behavior. All the data used in the system control and modeling, as well as, the data for the system design, were obtained through tests carried out in the LEEQE prototype of a water pumping system. Thus, the methodology of the PLC application and the development of a supervisory system in real time monitoring and definition of controller parameters are presented.

The main contribution of this work is presented in the use of BELBIC built in PLC applied to an industrial flow control system, showing the responsiveness of this type of controller in a real industrial environment.

The BELBIC designed here considers a digital controller based on the discrete-time transfer function. In addition, BELBIC parameters were adjusted using a PSO.

The paper is organized as follows. Section 1 presents the LEEQE water pumping workbench. In section 2 a modeling of the water pumping work bench using a transfer function will be approached. Section 3 describes the structure of the BELBIC-PSO controller, and then its implementation in the computational and PLC environment. Finally, conclusions are given in section 5.

## Industrial pump system

**Figure 1:**
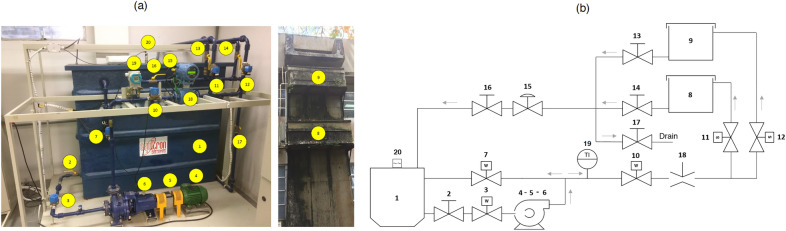
Water pumping stand prototype located at LEEQE. (**a**) Photography and (**b**) Diagram—created using PowerPoint software version Microsoft 365.

The experiments described in this article were performed at the LEEQE at UFPE. Currently the workbenches in this lab serve as the basis for advanced studies of industrial automation and control systems. Figure 1 shows an image of this industrial water pumping system.

A description of a pump system used in this study can be seen in Table 1:

**Table 1:** Equipment that compose the pumping prototype.

Number	Equipment	Number	Equipment
1	Water tank	11	On-off valve
2	Manual valve	12	On-off valve
3	Control valve	13	Mechanical valve
4	Induction valve	14	Mechanical valve
5	Torque and speed transducer	15	Mechanical valve
6	Centrifugal pump	16	Mechanical valve
7	Control valve	17	Drain valve
8	Resevoir 3 m height	18	Electromagnetic flowmeter
9	Resevoir 5 m height	19	Pressure transducer
10	Control valve	20	Sonar level transmitter

### Pumping system modelling

The model that will be developed in this paper will be obtained from the data extracted from the pumping workbench which goal will be to represent the relationship between the motor rotation frequency, $U(s)$, and the water flow rate in the main line, $Y(s)$, through a transfer function, $G(s)$. Thus, a black box modeling will be used, since this model will be obtained only through the experimental data of this process^31^.

The identification of the dynamic system is an important part for the realization of the application of BELBIC in the computational environment of Simulink MATLAB. The model representation will serve as a reference parameter for the system behavior in a real environment, since the proposed controller model will be implemented in the PLC.

The LEEQE pumping workbench set can be operated with frequencies between $0$ and $60Hz$. However, for values bellow $20Hz$, that defines the water flow rate in the main line of approximately $35m^3/h$, the pressure applied by the water column added to the atmospheric pressure, prevents the motor pump set from being able to pump water into external reservoirs, a common phenomenon to pumping systems known as cavitation. Thus, the operating range of the pumping system is defined between $20Hz$ and $60Hz$ establishing the main line flow rate of approximately $2400m^3/h$.

For the experiments that will be performed in this paper, we chose to work in a frequency range that is within the limits of the operating range of the system, where its behavior had no limitations in operational conditions, thus, the values defined as the minimum value of $37,5Hz$ is applied to the motor pump set, which sets a flow rate of approximately $1100m^3/h$, a maximum value of $53,5Hz$, which sets a flow rate of approximately $2200m^3/h$.

To simplify the process of the modeling real systems, the aspect of linearity can be considered, satisfying the superposition principle. Assuming that, in a given system when applying an input $u_1 (t)$, a $y_1 (t)$, output is produced, when applying an input $u_2 (t)$, a $y_2 (t)$, output is produced, this system satisfies the superposition principle if performed by $\alpha u_1 (t) + \beta u_2 (t)$ and its output is $\alpha y_1 (t) + \beta y_2 (t)$, in which $\alpha$ and $\beta$ are constants. Another system maximum operations frequency issue which must be taken into account is the invariance in time. A system is said to be invariant in time if a time offset in the input causes a time offset in the output. If $u(t)$ and $y(t)$ are respectively the input and output of a system, it will be invariant in time if $u(t-t_0)$ produces $y(t-t_0)$^32^.

As the pumping system will not have its pipe dimensions changed or any other components replaced during all the experiments to be performed, it will be considered invariant in time.

Regarding the linearity of this process, it was decided to investigate the behavior of the water flow rate by making specific changes in the system input, i.e., the motor frequency. A common procedure in these cases is to analyze the response of a given system by applying specific stimulus signals, such as the step function, which is used to determine the predominant dynamics of a given process^32^.

For this, other two frequency values were chosen, within the minimum and maximum ranges established: $42,3Hz$, which establishes a flow rate of $1400m^3/h$, and $47,8Hz$, which establishes a flow rate of $1800m^3/h$. This way,a three step input will be applied to the open loop system, so that three transfer functions are estimated and analyzed in relation to the variability of their parameters. The relation between all frequency and flow values can be seen in Table 2.

**Table 2:** Relation between frequency and flow values used for estimation of the transfer functions.

Frequency ($Hz$)	Flow rate ($m^3/h$)
37,5	1100
42,3	1400
47,8	1800
53,5	2200

**Figure 2:**
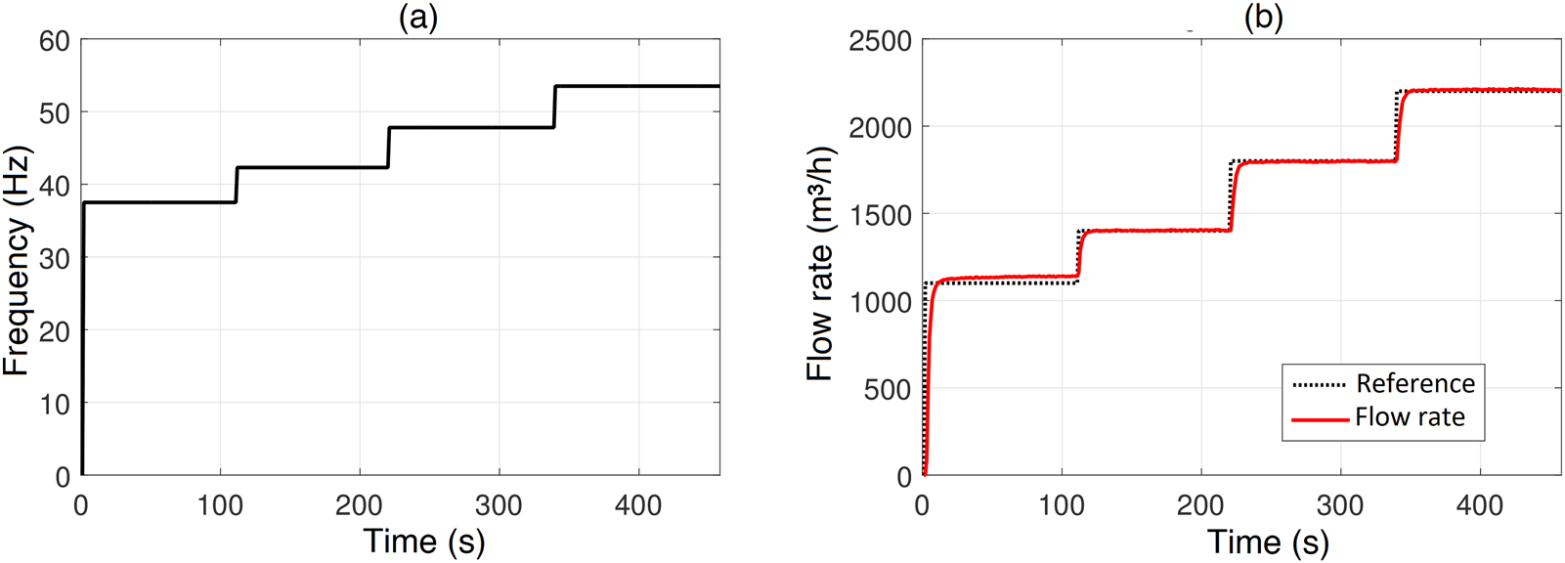
Experiments, curves, using the open loop pumping system to analyze a relation between motor frequency type changes and leakage variation.

This experiment used the following methodology: it was defined a 100% opening of the main line valve and water intake valve; only the external reservoir at a height of $3m$ will be used, which implies opening the inlet and outlet valves of this reservoir and closing the inlet and outlet valves of the external reservoir at a height of $3m$; with the system idle, allows the motor pump assembly to be started by the frequency inverter up to $37,5Hz$; after around 2 minutes, another step variation will change the frequency value to $42,3Hz$; this procedure will be repeated for the other two frequency values, always for an estimated period of 2 minutes. The flow rate signal was acquired with a sampling rate of 1 second, through the supervisory system and can be seen in Fig. 2a, b.

The analysis of the experimental results, represented in Fig. 2a, b, allows us to imply that the flow rate variation, regardless of the input value applied by the variation of the motor rotation frequency, behaves as a first-order system, that is, without the presence of a overshooting in the signal related to the reference values. Systems of this order are characterized by their time constant, defined as the time it takes the system to reach approximately 63.2% of its final value, when a step signal is applied to its input, and by the proportional gain that the input signal is processed^32^. Thus, the transfer functions estimated for the steps shown in the Fig. 2.

**Table 3:** Transfer functions estimated.

Input signal variations	Estimated transfer function
$Step~2: 37,5-42,3 Hz$	$G_1(s)= \frac{33,1362}{1+1,7091s}$
$Step~3: 42,3-47,8 Hz$	$G_2(s)= \frac{37,5965}{1+2,5861s}$
$Step~4: 47,8-53,5 Hz$	$G_3(s)= \frac{41,2874}{1+2,8085s}$

**Figure 3:**
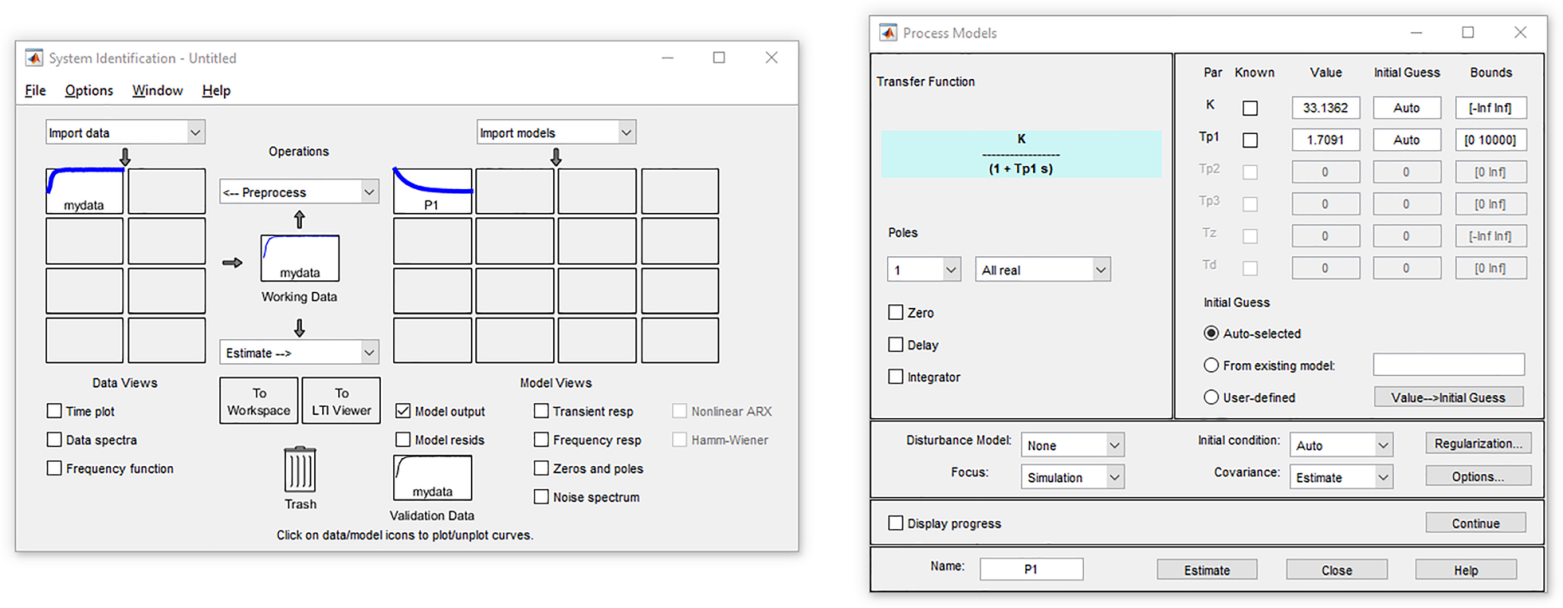
MATLAB system identification toolbox.

The system identification tool that will be used to set the values of these transfer settings will be the MATLAB System Identification Toolbox, through its graphic interface *Ident* (Fig. 3). The estimated transfer functions, accounts for the steps variation applied to the system presented in Table 3.

As showen, the three transfer functions found are different regarding the gain and time constants, which characterize the non-linearity of the pumping system in its operating points. Therefore, a single controller with fixed parameters should not be used for the entire operating range, as it would not be able to guarantee the same performance^33^.

The model used is close to the real process, which cannot fully incorporate all the characteristics of the real system. There must be a relationship between the cost of having the model and its level of detail in terms of the benefits expected from its application^34^.

The identification of the plant model and its transfer function were carried out experimentally from a set of experiments in the physical system of the pumping system, which made it possible to obtain the input-output pairs of the system in open loop. The analysis carried out in this work focused on the control of the water flow through the frequency inverter, from which the process input signal is a frequency value in the motor, regulated by the frequency inverter.

### Digital modeling

Although most controller design techniques are in continuous time, their implementation takes place in digital format (subject to discretization and quantization effects). These effects must be considered when tuning the controller, as they significantly affect the controller’s behavior.

For implementation of the BELBIC controller, the MATLAB toll Simulink was used, this tool can be used for modeling, simulating and analysing linear and nonlinear dynamic systems, continuous and / or discrete in the time. In addition to providing a graphical modeling environment that includes predefined block libraries and an interactive graphical editor for assembling and managing block diagrams, its simplicity makes it easy to modify the model, making it quick and easy to compare results.

Most systems in the area of process control and automation are based on microprocessor electronics, so they work in discrete time. The replacement of a continuous time controller for a discrete time controller will result in the same performace of the control task. Therefore, the difference is related to discrete control signals, which work with samples of signals detected at a given time, rather than continuous signals.

However, most techniques in controller designs are done in continuous time, but their implementation is in digital format, which will be subjected to discretization and quantization effects. Thus, discretization in the tuning of the controller should be taken into account, as it significantly affects the behavior of the controller^35^.

For an approximate match between continuous and discrete systems, a part of the system (continuous plant and the controller output) is considered; the remodeling process is in accordance with the Zero-Order-Hold discretization technique, since the actual process dynamics is influenced by the data sampling rates.

**Figure 4:**
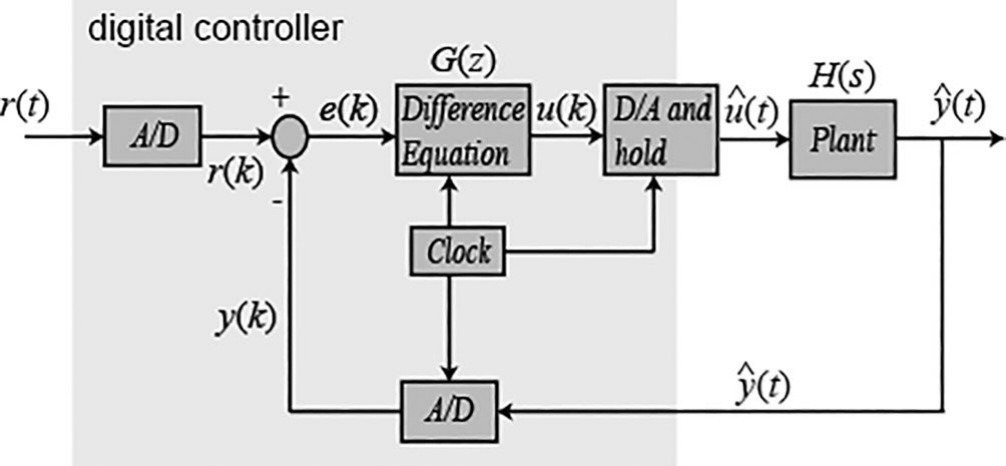
Equivalence of the continuous-digital process in a control mesh^36^.

In order to find the digital model for the linear part of the system transfer functions described in Table 3 we used the $c2d$ (continuous to discrete) command in the MATLAB environment to apply the Zero-Order-Hold method to these equations. The sample rate chosen for the discretization of the continuous time transfer functions was 1 second; the choice was mainly due to the reaction of the plant dynamics, because the flowmeter in the main line of the water pumping system provided data in this time interval. Figure 4 below illustrates the discretization process of transfer function by the Zero-Order-Hold method.

By choosing an inaccurate sampling time in the $H_zoh(z)$ discretization it will result in significant errors, because unlike the continuous system, it is known that the discrete system performs operations in the same sampling rate of the time intervals, and information may be lost, or even overshoot error can occur^36^.

Subsequently the discrete time transfer functions are shown—Eqs. (1), (2) and (3)—in relation to Table 3, respectively, using the Zero Order Hold method, with a 1 second sampling time.

1 $$\begin{aligned} H_1(s)= &  \frac{14,68}{z-0,557} \end{aligned}$$

2 $$\begin{aligned} H_2(s)= &  \frac{12,06}{z-6793} \end{aligned}$$

3 $$\begin{aligned} H_3(s)= &  \frac{12,37}{z-0,7004} \end{aligned}$$

## Methodology

This work presents the implementation of the BELBIC controller in a PLC for the flow control of the LAMOTRIZ industrial pumping system, however the idea of this controller can be generalized to several industrial processes. Initially, the system was simulated in a MATLAB computational environment to analyze its behavior. Then, the PSO technique will be used to calculate the parameters of this controller.

Thus, in addition to presenting the application methodology in the PLC, the development of a supervisory system for real-time monitoring and definition of controller parameters will also be presented. Finally, the closed-loop tests of this controller are performed with the respective changes in the flow reference values, and then the robustness analysis of the BELBIC controller in relation to the variation of the operating conditions of the system. In this case, simulating some random water consumption scenarios through a water recirculation valve located in the main line of the pumping system.

### BELBIC

Since the purpose of this article is an application in control systems, no biological concepts, not all system structures will be presented. Therefore, the study will focus on the main structures that influence the development of the proposed model.

Many systems have nonlinear characteristics and even time variations that interfere directly with control systems, such as deterioration of the component or variations in environmental parameters that impair significantly the performance of control systems. For this reason, strategies that modify the controller structure or simply its parameters have been increasingly used. Such strategies are independent of the complexity of control laws, as can be seen in^37^, where several proposals for the use of adaptive PID controllers are applied to nonlinear systems.

The evolution of so-called intelligent control systems, named this way for emulating particularities of human intelligence and its learning ability, has allowed these types of controllers to be a very efficient alternative, as it is the case with artificial neural networks (ANNs)^38^.

Intelligent systems take into consideration their ability to learn and adapt parameters in order to improve system performance and overcome the difficulties encountered with environmental changes^39^.

**Figure 5:**
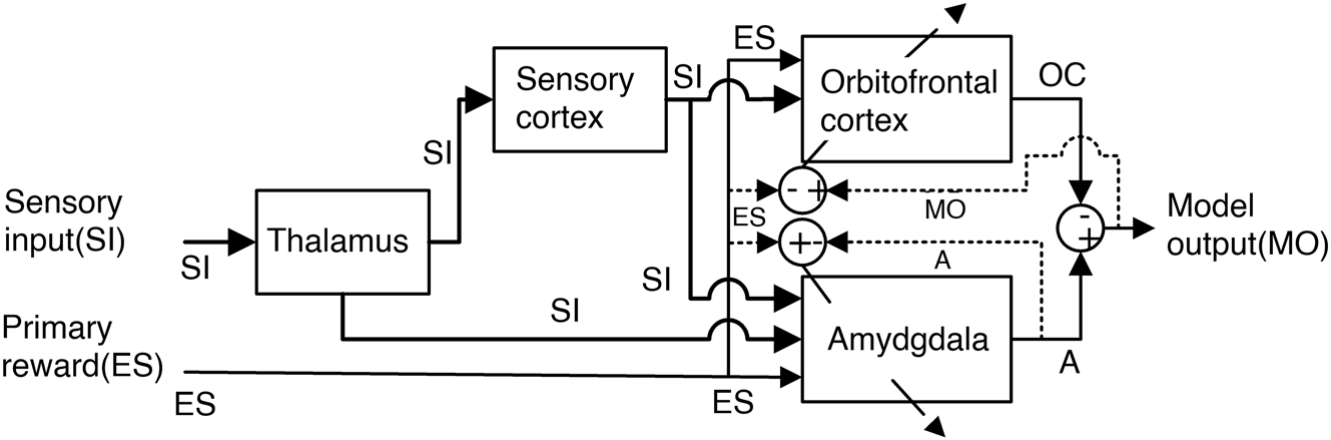
Computational model of the amygdala emotional learning—created using PowerPoint software version Microsoft 365.

Motivated by the success of the functional modeling of emotions in control engineering applications, a structural model (Fig. 5) based on the limbic system of the mammalian brain was developed. The model consists of two main areas, orbitofrontal cortex and amygdala, which are responsible for the achievement of learning algorithms. Thus, the development of this model will have an intelligent system with the ability to learn quickly on decision making, which is very effective in applications on control engineering^8^.

The limbic system responds to instinctive behaviors, thoughts and ways of being, including our personality, reactions to external stimulus, memory, basic impulses, anger, pleasure and survival, in addition to the functions mentioned, according to^40^.

**Figure 6:**
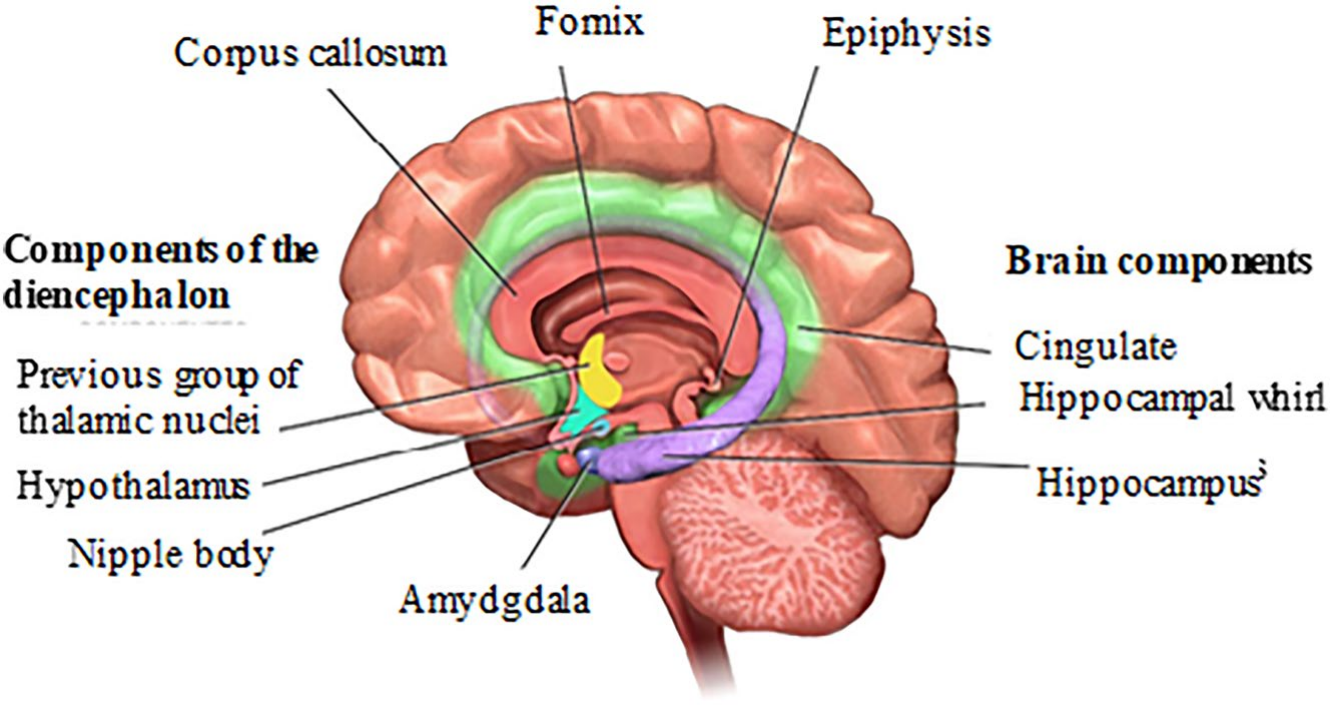
Anatomical view of the brain limbic system^43^.

As it is well known, there is no agreement among all the authors that brain structures form the limbic system. The first neurologist to link some brain structures to emotions was the Frenchman Pierre Paul Broca (1877) describing the “great limbic lob”^41^. He noted that the amygdala, hippocampal formations, and cingulate turns were all related to emotions. Broca was the first scientist to call all these structures located around the diencephalon “the great limbic lobe” (Fig. 6); in the medial region of the cerebral hemispheres. The term limbic was also adopted because of its meaning (from Latin Limbus: edge, ring, around), confirmed by Sarnat and Netsky, since these structures, present in all mammals, are located around the top of the brainstem^42^.

As the focus of the article is the application in control systems and not biological concepts, the established model will not include all limbic system structures, only the modeling of the following structures: amygdala, orbitofrontal cortex, sensory cortex and thalamus. In fact, a computational mathematical model will be presented by a set of equations which quantitatively define the phenomenon.

A key feature of the model is the fact that the motivation to respond, and the response itself are different^44^, thus allowing a vast pattern of responses to external stimuli. Thus, the stimulus assessment and the choice of actions to be taken as a result of the assessment are clearly separate. The motivation for this statement comes from biology, where the task of the amygdala is to learn the associations between sensory and emotional input and to reflect them on the output^45,46^.

Still according to this statement, the trend of amygdala learning is monotonic, that is, it can only increase^47^. Whether the experience is favorable or unfavorable, the amygdala captures the essence of this association and tends to function as the basis for future new experience. But the final action generated by the limbic system is still controlled by the orbitofrontal cortex (OC—orbital cortex). In this context, there is a shortcut path between the thalamus and amygdala (A) which will be responsible for improving the model’s speed and tolerance to failure of the model, as it ignores the processing of the longer sensory cortex. Thus, this shortcut allowed the model to generate a quick (though not optimal) action called satisfactory decision. In addition to carrying as much as possible information within the multiple sensory inputs, in case of malfunction of the sensory cortex due to an excess of contradictory sensory signals.

**Figure 7:**
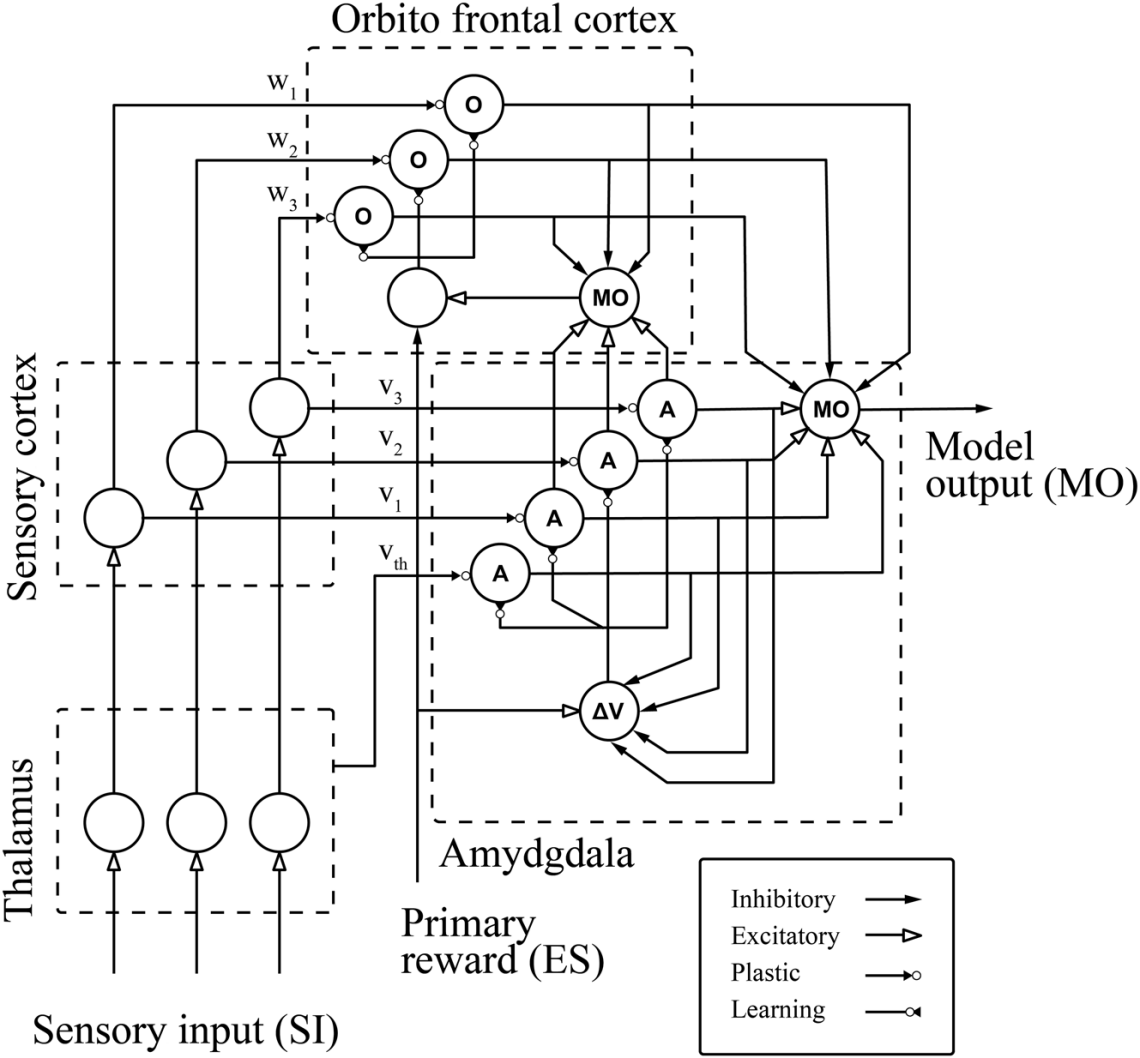
A graphical depiction of the brain emotional learning process^8^.

Figure 7 presents a computational model of the interaction between the amygdala and orbitofrontal cortex in an emotional conditioning described in^8^.

The system consists of four main parts. Sensory input $(SI)_i$ signals first enter the thalamus where preprocessing is performed and then these signals are sent to the sensory cortex and amygdala. The sensory cortex is responsible for the subdivision and unrefined distinction of the thalamus outlet. Later these signals are sent to the amygdala and orbitofrontal cortex^48^.

On the other hand, in the phase that happens in the amygdala, the signal will undergo an emotional assessment of the stimuli. This assessment is in turn used as the basis of the emotional condition. Finally, the orbitofrontal cortex is tasked with inhibiting inappropriate amygdala responses^7,8^.

To obtain the equations that will represent this system, we adopted the amygdala signal as $(A)$ and the orbitofrontal cortex $(OC)$ . This way, for each sensory input received by the model $(SI_i)$. There is a corresponding node in the amygdala $(A_i)$ and also, in the orbitofrontal cortex node $(OC_i)$, which generate the nodal outputs of the amygdala and orbitalfrontal cortex. Therefore, these outputs are generated by the product between the sensory input signal and by their corresponding amygdala $(V)$ and orbitofrontal cortex $(W)$ weights, resulting in:

4 $$\begin{aligned} A_i= &  V.SI_i \end{aligned}$$

5 $$\begin{aligned} OC_i= &  W.SI_i \end{aligned}$$

It is the thalamus’s task to provide a non-optimal, but rapid response to stimuli. This ability will make it surpass the maximum signal between all sensory inputs $(SI_i)$ and send it to the amygdala as an input $(A_{th})$^7,49,50^.

6 $$\begin{aligned} A_{th} = max(SI_i) \end{aligned}$$

The equations that are set up with the index $(i)$ imply that their emotional processing has multiple loops and all individual loop outputs will result in a single output $(MO)$ model. The blocks of the amygdala and orbitofrontal cortex basically have adaptive weights that act on $(SI)$ and these weights are updated by $(\Delta V)$ and $(\Delta W)$, depending on the emotional signal and other signals. The learning process of the amygdala and orbitofrontal cortex occurs through its internal rules for weight update given by Eqs. (7) and (8):

7 $$\begin{aligned} \Delta V= &  \alpha .SI_i.max(0,ES-\sum _iA_i) \end{aligned}$$

8 $$\begin{aligned} \Delta W= &  \beta .SI_i.R_o \end{aligned}$$

The value of $(\alpha )$ is fixed and used to adjust the learning speed, on the other hand $(\beta )$ is the learning rate. The weight $(V)$ will not decrease, because of the learned emotional reaction, it will be lasting, and the task of the orbitofrontal cortex is to inhibit this reaction when it is inadequate. The learning rule of the orbitofrontal cortex is very similar to the amygdala’s, but its weight $(W)$ may increase or decrease as necessary to track the needed inhibition.

The term $(max)$ of Eq. (7) makes the changes in learning monotonic, in such a way that the amygdala gain will not ever decrease. This rule implies, that once modeled in the system, incapacity of deactivating the emotional signal (and, consequently, the emotional action) previously learned in the amygdala^51^.

Given the Eq. (8), $(R_o)$ is defined as internal reinforcement for the orbitofrontal cortex, represented by the following equations, in which the emotional signal $(ES)$defines the result of $(R_o)$ calculation.

9 $$\begin{aligned} R_o \left\{ \begin{array}{ll} max(0,\sum _i A_i-ES)-\sum _iOC_i ~~~~~~~~~~~ &\forall ~~ES\ne 0 \\ \\ max(0,\sum _i A_i- \sum _iOC_i ) ~~~~~~~~~~~ &\forall ~~ES = 0 \end{array} \right. \end{aligned}$$

Thus, in the presence of a reward, the internal reinforcement $(R_o)$ , represents the discrepancy between the rewards and the amygdala outputs $(A_i)$ subtracted by the orbitofrontal cortex output $(OC_i)$. However, if there isn’t a reward, the cortex behaves in a different manner, in which the $(R_o)$ will be the excess of the amygdala outputs over the cortex outputs $(OC_i)$ , as show in the Eq. (9).

The achievement of the model output $(MO)$, common to all model outputs, is simply the diference between the sum of the outputs ($A_i$—excitatory exits) and ($OC_i$—inhibitory outputs), respectively, thus the result of the model is presented by Eq. (10).

10 $$\begin{aligned} MO= \sum _iA_i - \sum _iOC_i \end{aligned}$$

The first task in the use of the model for a control system application is a way to incorporate it in the global architecture of the system, in which there is not a single way of doing it. An important characteristic of the BELBIC controller is its flexibility to receive different sensorial stimuli and emotional signals. This controller has many parameters which allow freedom to choose what suits a better response.

In the field of systems control engineering, the S and R signals provide the BEL module with the ability to make it noticeable to dynamic system changes and contribute to achieving the control objectives. About the design of the BELBIC controller, the *SI* is associated with the speed and gain of the dynamic response, on the other hand, the *ES* is closely related to the performance dynamics of this controller.

In the literature, the composition of SI and ES is at the discretion of the designer, based on knowledge of the plant’s dynamics and experimental tests, it’s possible to obtain such sensory and emotional signals. In this sense, it’s possible to choose a set of different architectures for both signals, such as the different variables of the control loop in which the BELBIC is inserted.

According to works related to topic^52,53^, it is noted that the proper definition of sensory and reward, is that they can promote the correct and proper functioning of the BELBIC in control engineering is not a simple task. Therefore, it is necessary to correctly understand the effects of both signals (SI and ES) on the final result of the BELBIC control and, in addition, the plant control variables that will form the architecture of these signals. All these aspects make the modeling of these signals an iterative process, containing several tests and adjustments to determine the best values attributed to the gains, which control variables should or should not be involved in the composition of the signals.

**Figure 8:**
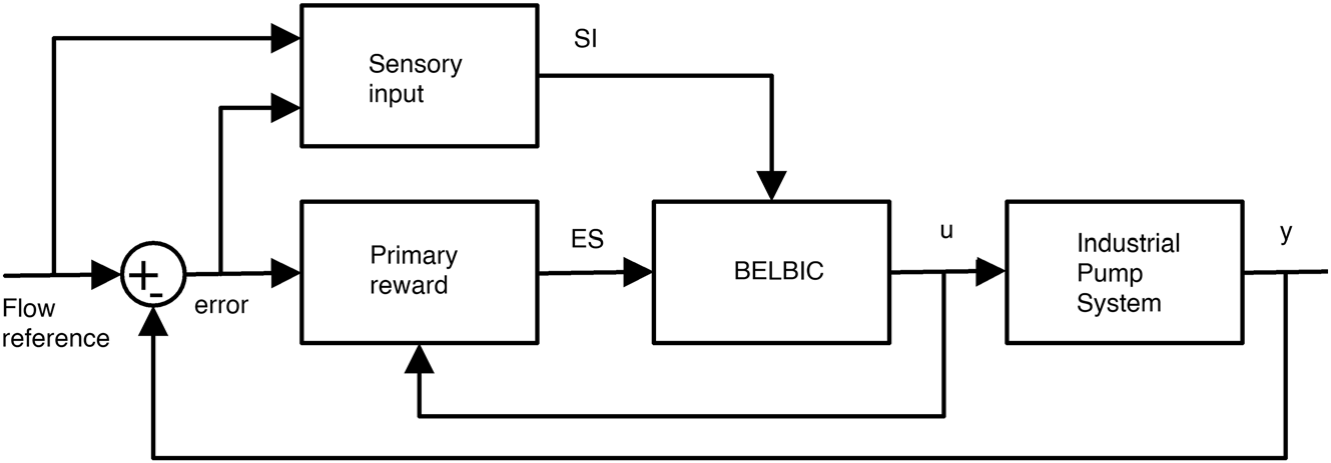
Block diagram of *SI* and *ES* inputs—created using PowerPoint software version Microsoft 365.

Therefore, a possible candidate, in this case, to incorporate the BEBLBIC, is presented in Fig. 8.

The block diagram (Fig. 8), represents the typical feedback control, which assumes the emotional signal $(ES)$, sensorial stimuli $(SI)$, controller output $(u)$ and creates a $(error)$ signal, which is the difference between the reference flow rate and the plant output $(y)$.

With regard to the controller operation, the $(ES)$ is the weighted combination of the error, its integral and the control action. As expected, there is a flexibility to generate the emotional signal choosing the emotional inclinations that can implicitly decide the control goals.

The sensorial response chosen as the combination of the error integral and its reference value in the control mesh, makes the system more sensitive to its status change. Thus, the obtained equations were Eqs. (11) and (12) for the sensorial input and Eq. (13) for the emotional signal.

11 $$\begin{aligned} SI_1= &  K_4.\int e dt \end{aligned}$$

12 $$\begin{aligned} SI_2= &  K_5. Ref \end{aligned}$$

13 $$\begin{aligned} ES_1= &  K_1.e+K_2.\int e dt +K_3.u \end{aligned}$$

The related terms that make up the equations are: the tracking error $(e)$, the reference signal of the flow rate desired value $(Ref)$ and the BELBIC controller output signal $(u)$. In addition to that, the parameters $K_1,K_2,K_3,K_4 e K_5$ are the associated weights to the signals previously mentioned. These parameters $(K)$ are initially empirically estimated, based on simulations and, posteriorly, an optimization technique based in particle swarm will be used to optimize the values of these parameters.

### Particle swarm optimizaion

In order to perform a better parameterization of the BELBIC, it was chosen in this work to use the metaheuristic algorithm of particle swarm optimization (PSO). In general, the PSO presents a satisfactory performance in the search for correlational parameters, obtaining results in times that are generally shorter than traditional optimization algorithms. it does not use the gradient of the problem being optimized. In other words, unlike traditional optimization methods, PSO does not require the problem to be differentiable. Furthermore, an important advantage in using this method is the presence of few hyperparameters. For the same hyperparameters, PSO will work on a wide variety of tasks, which makes it a very powerful and flexible algorithm.

The PSO is a stochastic optimization technique^54^, based on the social and cooperative behavior exhibited by many species to fulfill their need in the search space. The algorithm keeps a swarm of particles, where each particle represents a possible solution. The particles are casted in a multidimensional search space, in which the position of each particle is adjusted according to their own experience and their neighbors.

The first step of the algorithm is to create an initial population (Eq.  14) of size $(N)$ and dimension $(D)$ and each particle (Eq. 15).

14 $$\begin{aligned} X= &  [X_1,X_2,\ldots,X_N]^T \end{aligned}$$

15 $$\begin{aligned} X_i= &  [X_{i,1},X_{i,2},\ldots,X_{i,D}] \end{aligned}$$

Besides that, the initial speed of the population (Eq. 16) and each particle speed (Eq. 17) are calculated:

16 $$\begin{aligned} V= &  [V_1,V_2,\ldots,V_N]^T \end{aligned}$$

17 $$\begin{aligned} V_i= &  [V_{i,1},V_{i,2},\ldots,V_{i,D}] \end{aligned}$$

The indexes satisfy the condition of Eq. (18):

18 $$\begin{aligned} i=[1~N]~~e~~j =[1~D] \end{aligned}$$

The particle speed is the central element of the entire optimization, and it is altered according to the relative positions *pbest* and *gbest*, best individual location and best global location, respectively. The particles are accelerated towards the locations of higher fitness according to Eq. (19):

19 $$\begin{aligned} v_{i,j}^{k+1}=wv_{i,j}^{k}+c_1rand()(pbest_{i,j}^{k}-X_{i,j}^{j})+c_2rand()(gbest_{j}^{k}-X_{i,j}^{k}) \end{aligned}$$

The $c_1$ (cognitive rates) is a determinative factor of how a particle is affected by the memory of its best location and $c_2$ (social rates) is a determinative factor of how a particle is affected by the rest of the swarm. The adopted parameters values of $c_1$ and $c_2$ were 2, for both of them, as suggested by the PSO developers in order to maintain a balance over the influence of individual and social learning, in the current particle behavior^55^. The term w is called “inertial weight”, responsible for determining at which rate the particle remains throughout its original path affected by the *pbest* and *gbest* force. The further particles of *gbest* and *pbest* feel attraction of their respective locations, and this way, advance towards them faster. Realizing the importance of initial search exploration and the increasing importance of maximum search progression exploration, it was suggested that the value of the inertial weight would vary linearly between 0.6 and 1.2^55^. Since the speed was already set, it is an easy task to move each particle to their next position. New coordinates $x_n$ are calculated for each one of the N dimensions according to the Eq. (20):

20 $$\begin{aligned} x_{i,j}^{k}=x_{i,j}^{k}+v_{i,j}^{k+1}; ~~ \forall j ~~ e~~ \forall i \end{aligned}$$

**Figure 9:**
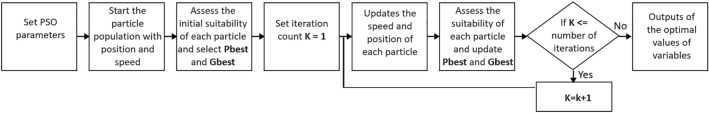
Flowchart of the PSO algorithm—created using PowerPoint software version Microsoft 365.

For the implementation of the optimization algorithm, it was used the computational environment of MATLAB. It is known that scripts can be typed in the command window and each line is processed immediately to simulate the PSO algorithm. The PSO flowchart is shown below in Fig. 9. Therefore, for the proposed application in this work the cycle continues until it meets the stopping criterion which is the maximum number of iterations. The application of this criterion should be taken with caution by choosing a reasonable number of iterations, because when this number is too high, the PSO may stagnate waiting for w to decrease to start the maximum exploration. Also, a small number of iterations can result in the exploration of local maximum even before the swarm can properly explore the solution space and find the highest global maximum.

### Controller parameter optimizations

**Figure 10:**
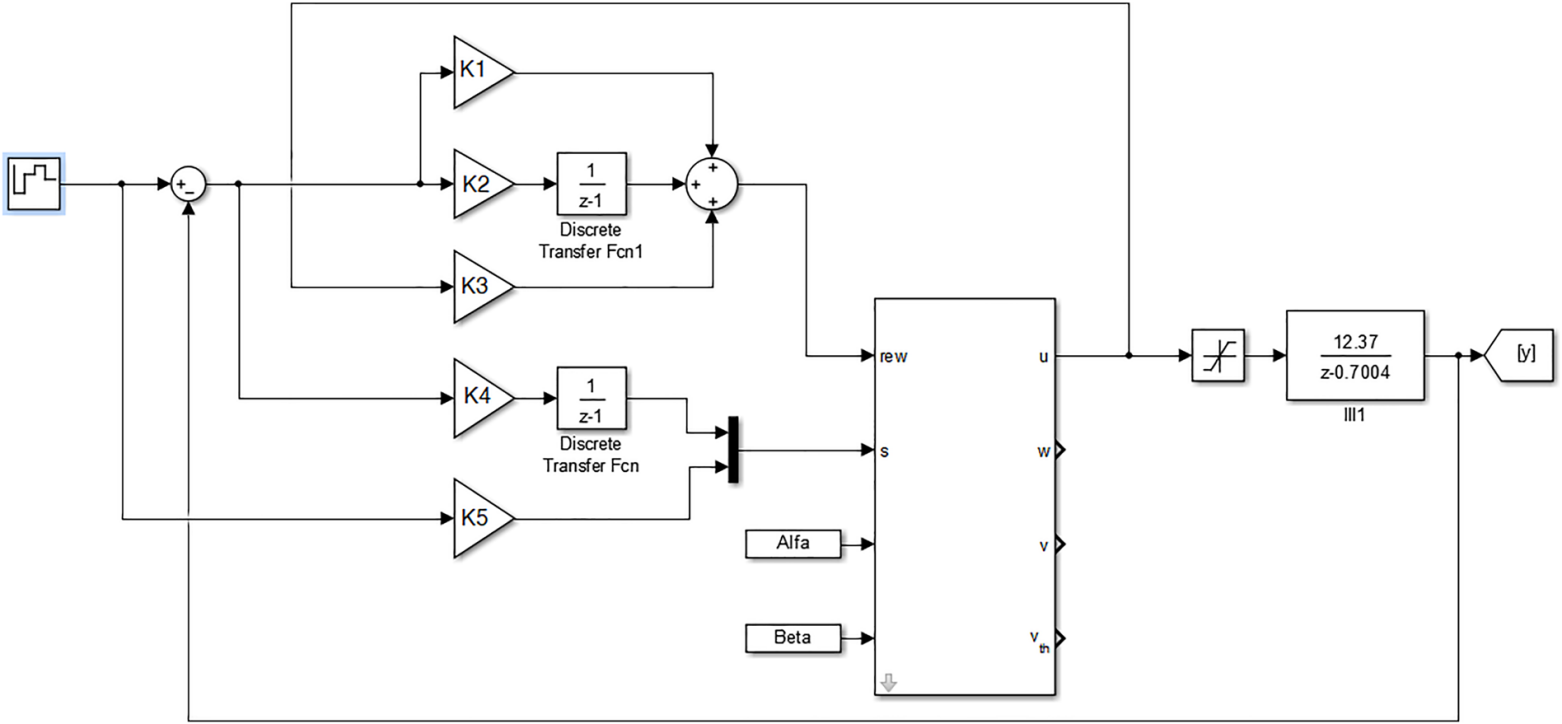
Control system block diagram with BELBIC controller.

For the majority of the optimization problems, having a prior knowledge of the solution search space makes the performance of the method more effective. By doing this, the chances of finding the ideal or very close solution increase considerably. This paper presents the implementation of PSO as a search tool for the adjusting parameters($K_1,K_2,K_3,K_4~e ~K_5$ represented in the Fig. 10) that will be used in the BELBIC controller. However, the authors themselves and the current literature, do not suggest any method of adjusting these parameters. The proposal was to implement the PSO to find the parameters that would suit each of the proposed experiments in this article. In a recent research^56^ the authors also used the PSO, but for the optimization of the parameters of a fuzzy controller.

Initially the BELBIC global structure was implemented in the MATLAB®computer simulator Simulink based on the controller equations defined in this article. In Paper^18^ we use the BELBIC approach to control an industrial fan system modelled in continuous system and here we are using a more sophisticated discrete system modelling. The modelling is based on an improvement of^57^ which shows a mathematical framework for both the continuous and discrete-time formulations and by presenting a Simulink computational tool. When developing all block diagrams referring to the limbic system structures, the developed block connections will be made to represent the closed loop system of the LEEQE water pumping system and the BELBIC controller. This pumping system, represented here by the transfer function, that defines the system plant, will serve to simulate the system behavior according to an operating range, with an alternation between the three transfer functions activated in Table 3. After completing the BELBIC controller connections. Figure 10 shows the closed loop control structure as shown in Fig. 8.

**Figure 11:**
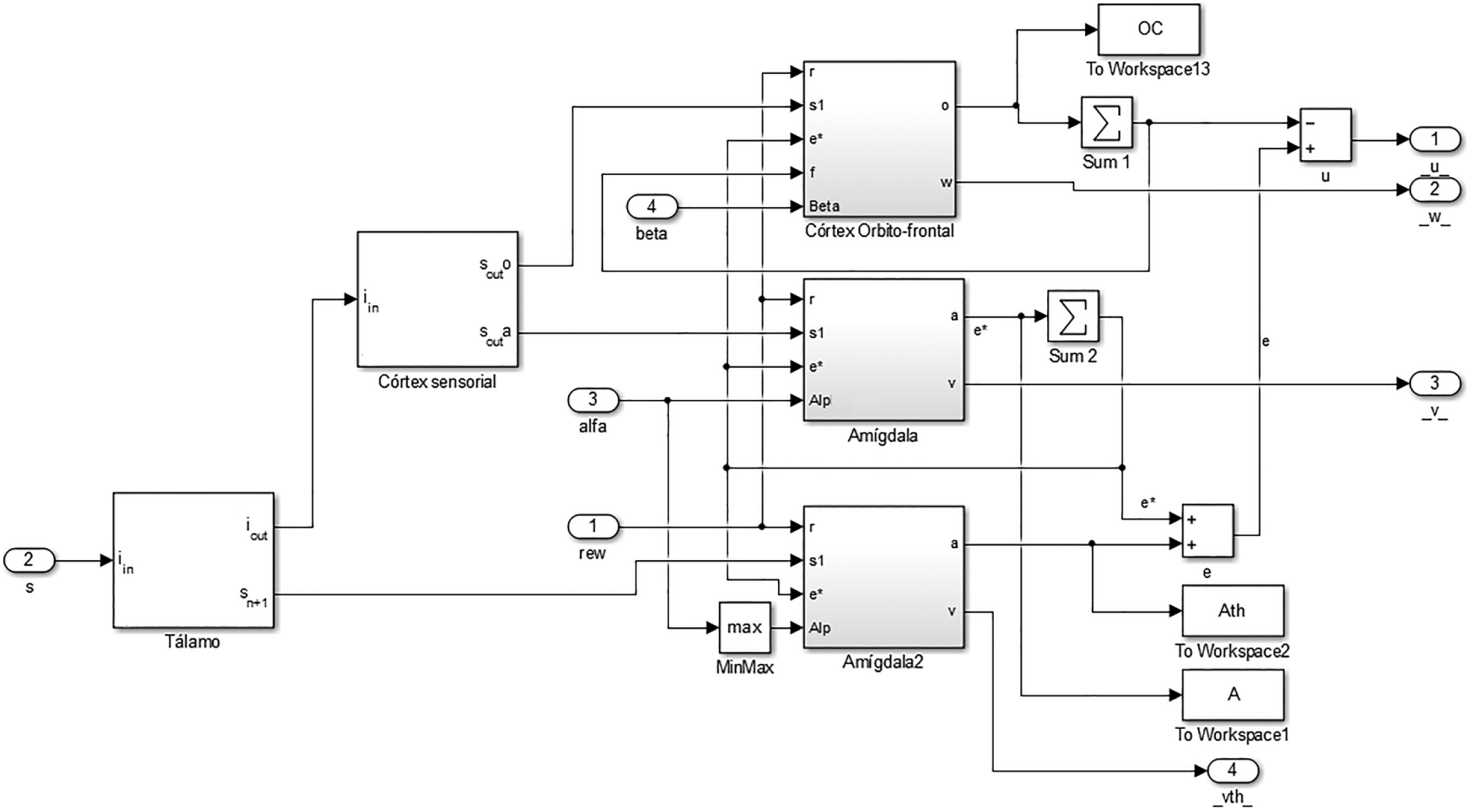
BELBIC controller block diagram.

In order to build the BELBIC controller and its respective connections, Fig. 7 and Eqs. (4) to (5) were used as the basis. Then defined the connections between the blocks of the respective limbic system structures proposed in^57^ were then defined to find the basic elements of the amygdala and orbitofrontal cortex and the BEL system as shown in Fig. 11.

The use of PSO in the estimation of the optimized BELBIC parameters requires modeling a function that best corresponds to the problem objective. To compose the equation, the performance index—Integrated Absolute Error (IAE) will be used. This index is one of the most used, mostly for obtaining good experimental results in computational implementation. A system design based on this criterion has as characteristic a reasonable damping, that is, it presents a good transient with low oscillating overshoot signal response. For the calculation of the IAE the following Eq. (21) is presented:

21 $$\begin{aligned} IAE=\int _0^T |e(t)|dt \end{aligned}$$

In relation to the analysis of discrete control systems, the integrals are expressed as summation and error as a function of discrete error *e*(*k*). Equation (22) represents the discrete time index.

22 $$\begin{aligned} IAE=\sum _{k=1}^N |e(t)| \end{aligned}$$

According^32^ the criteria based on the integral of the system error integrates a more classical way of evaluating the performance of a control loop, besides the parameters of the transient system response to the step input.

Given the established index, which is defined as a problem that needs to be optimized, the objective function (*FO*) Eq. (23), required here, is a minimization problem, composed by the IAE index.

23 $$\begin{aligned} FO(k)=\sum _{k=1}^N |e(t)| \end{aligned}$$

Its’s noteworthy that the stability analysis of the emotional controller by itself presents a high complexity, mainly because it is a non-linear control system. Several works presented proposals to analyze the stability of this type of controller. The work of^58^ , for example, presents a study of the stability of the emotional controller by Lyapunov, admitting in this situation an emotional controller structure as a non-linear system of universal approximation. In this work, although there is no study model for the stability of the emotional controller, the optimization process taken into account, previously, tested the operating limits.

### PLC application and supervisory control system

**Figure 12:**
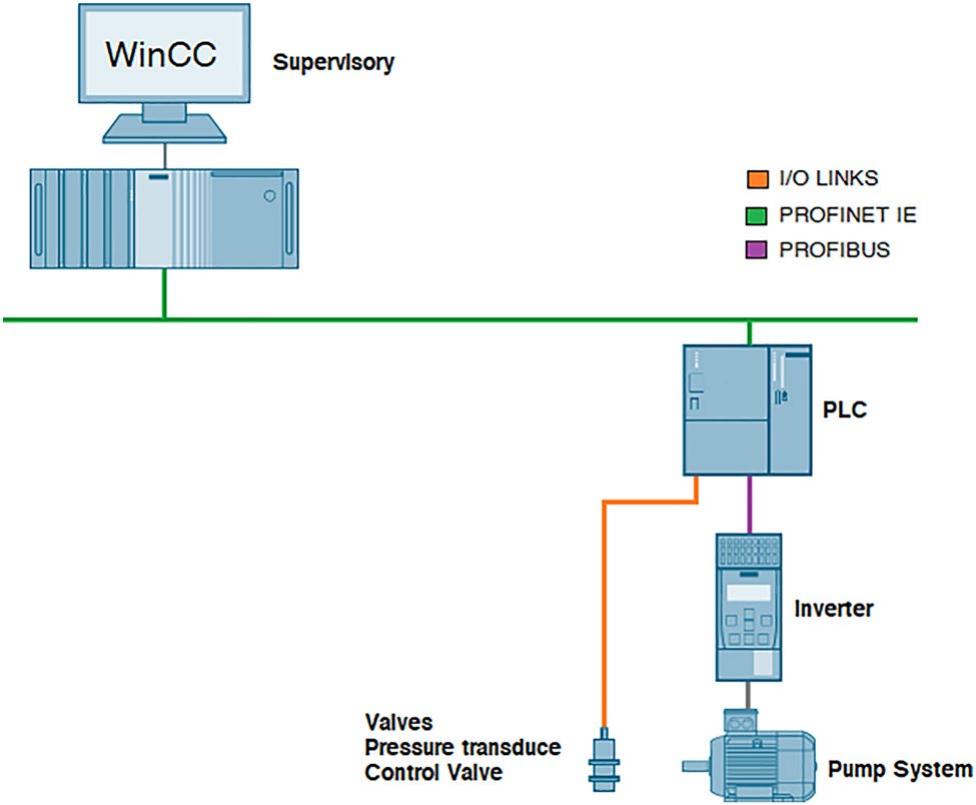
Pump bench topology—created using paint software version 20H2.

The pump bench network topology is shown in Fig. 12 . This structure allows the exchange of data between the supervisory system and the PLC. The PROFIBUS-DP network standard uses master-slave communication technology, which is capable of transmitting at high speed a large volume of information from the PLC device and the frequency inverter. In addition, the PROFINET network is also used in the presented topology, which in turn is based on an Industrial Ethernet communication standardized by the IEC 61158-5 and IEC 61158-6 standards. The laboratory represents the purpose of an industrial plant for a water pumping system, therefore the PROFINET network will access data in drives, I/O and workstations (Supervisory Systems).

The BELBIC PLC controller project was developed in STEP7 software using LADDER programming language and program structure in a partitioned way.

**Figure 13:**
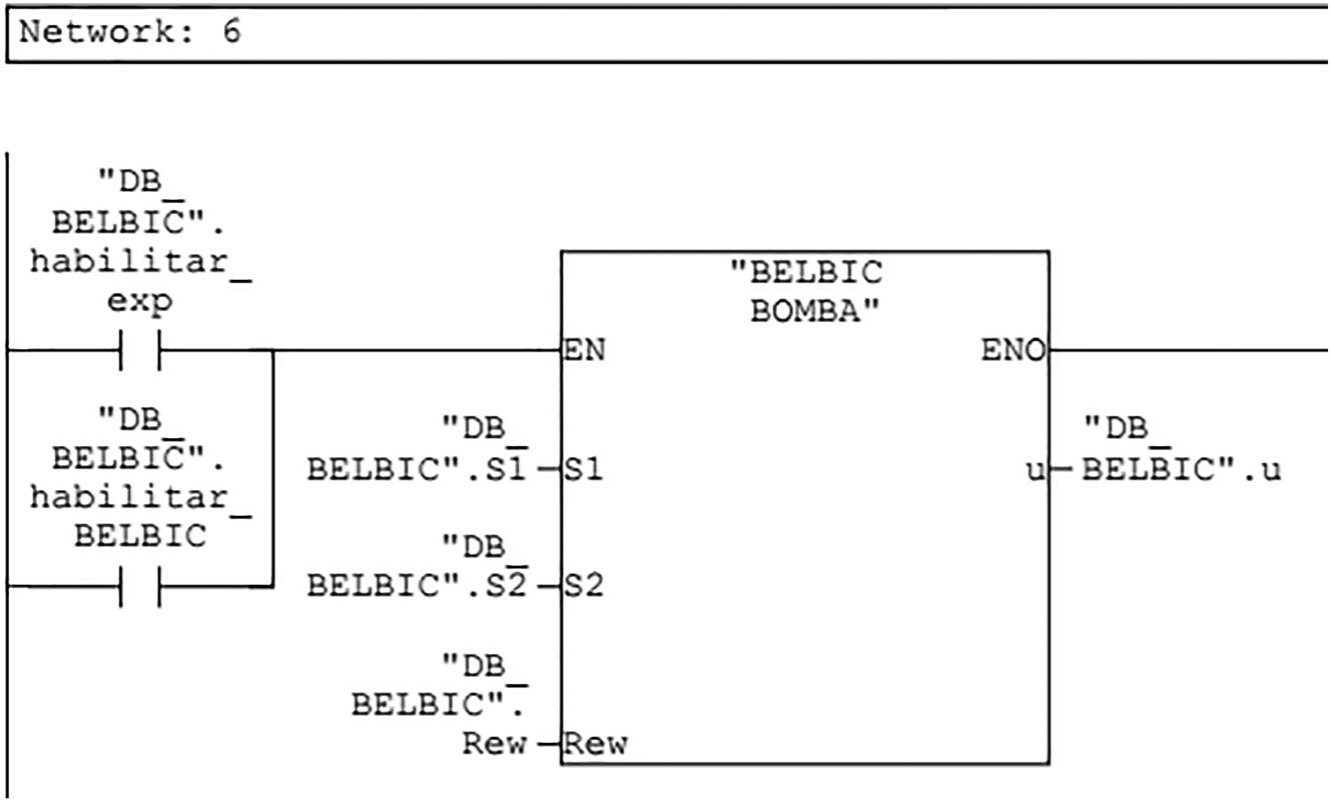
BELBIC controller PLC block.

Initially the block divisions were defined according to the structures of the limbic system. This smoothed the analysis of each block individually and the connections between them. By completing all the blocks required to develop the BELBIC controller, the blocks were clustered internally into a single block, shown in Fig. 13.

**Figure 14:**
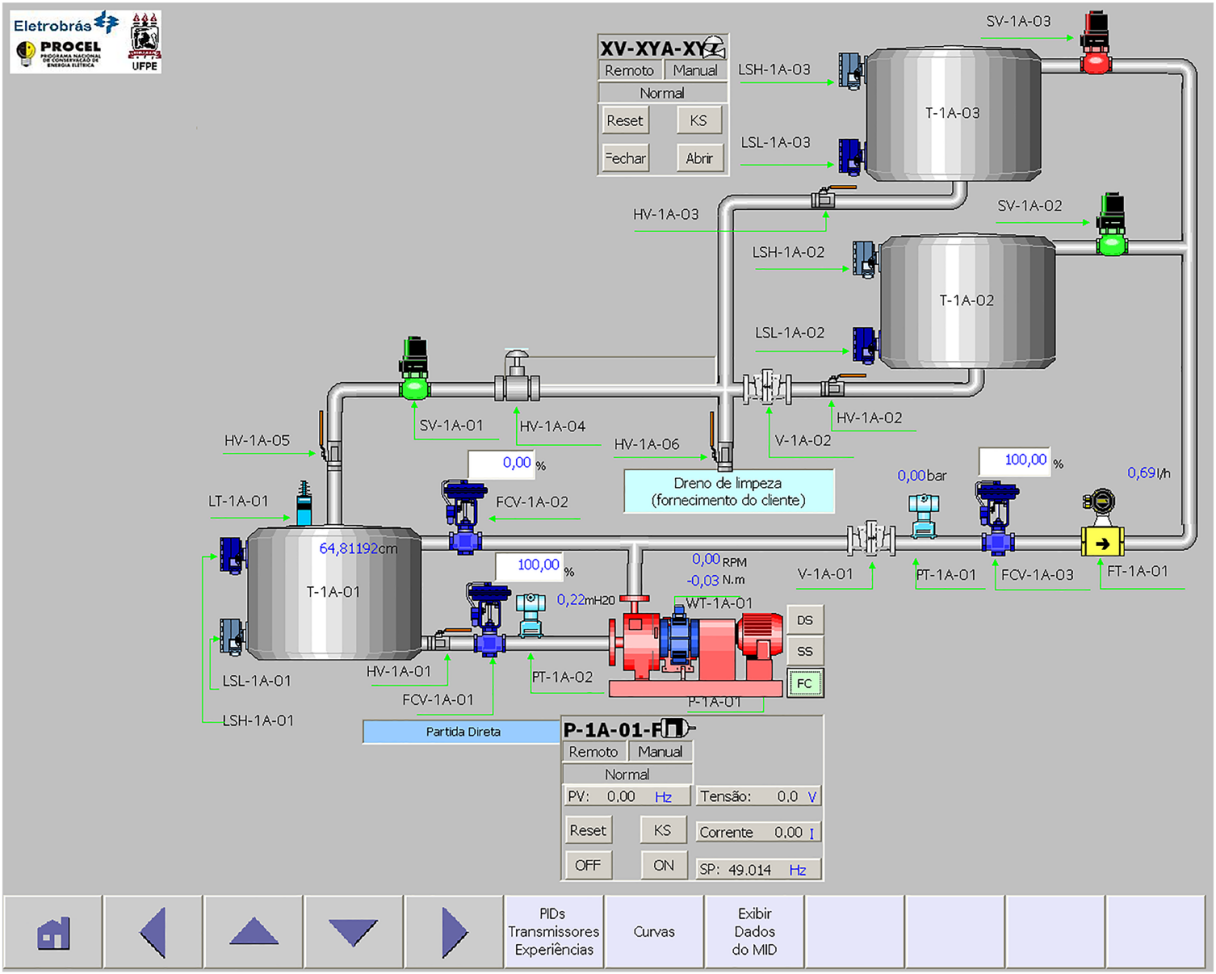
LEEQE pump bench preview screen in WinCC.

In many industrial processes, the use of supervisory systems is required to facilitate the human-machine interface, capture and store data, and perform process control tasks. Figure 14 shows the main screen developed using the WinCC Flexible software that represents the water pumping system used in this work.

To facilitate the access to all memories defined by the controller design developed in the PLC, and operation of this controller, it was necessary to create a supervisory system screen for the user monitor, store, change and analyze all data from the BELBIC controller.

**Figure 15:**
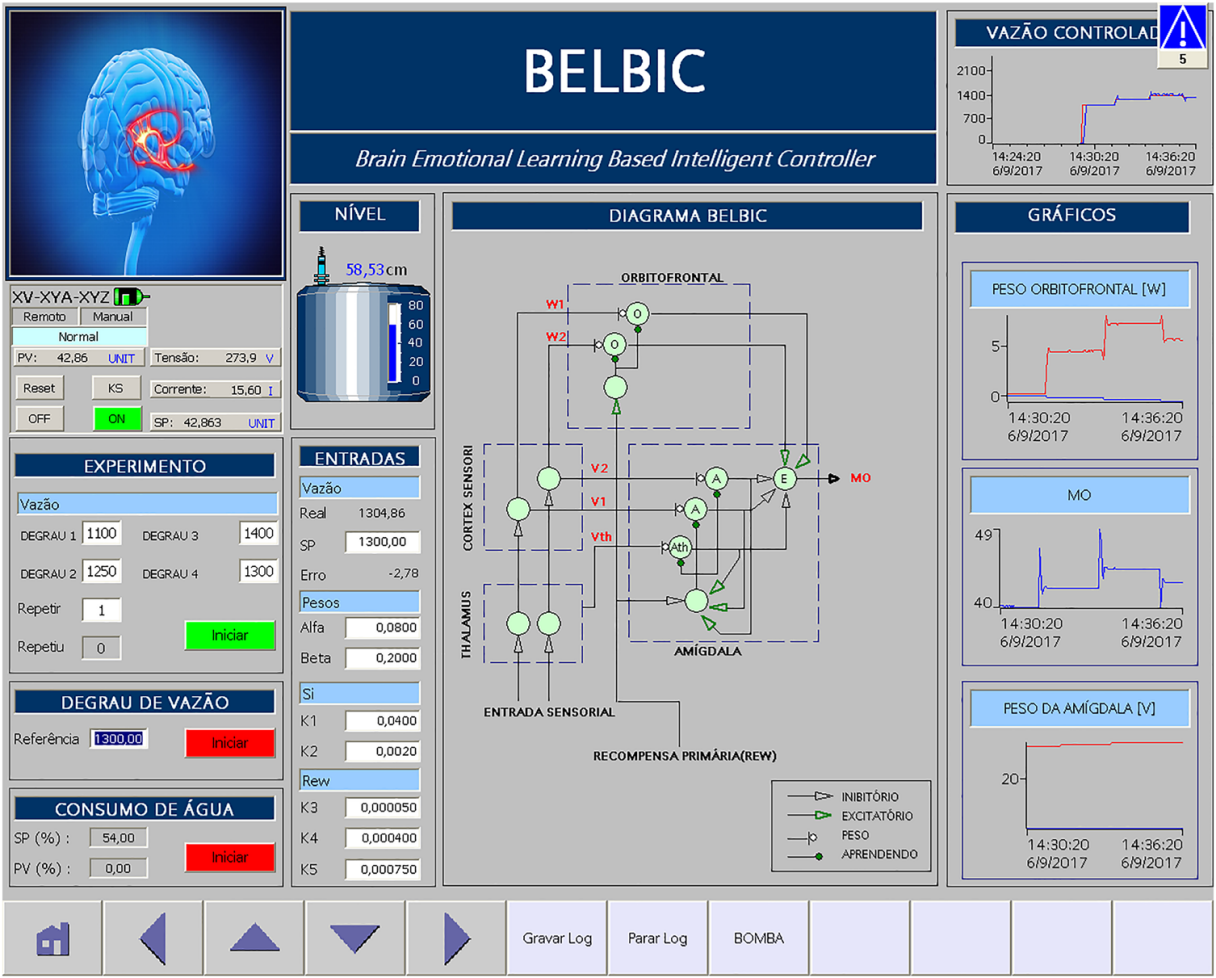
BELBIC controller supervisory screen in WinCC.

Figure 15 shows the BELBIC controller supervisory system screen. This screen consists of: buttons, figures, diagrams, graphs and fields for entering values (setpoints). The screen functionalities are: monitor frequency inverter data; define the desired flow rate setpoints for each experiment; observe the main line water flow values and the error obtained when using the controller; change the parameters of the BELBIC controller; graphically supervise some of the variables involved in the control process; randomly simulate water consumption for controller robustness testing and recording all desired variables in a database.

## Results

The BELBIC was applied to the pumping workbench causing the frequency inverter to act on the motor pump set up. Firstly, some flow rate values were established to implement the experiments according to the ranges mentioned in Table 2, thus, it was defined all the scenarios that will be controlled, as can be seen in Table 4.

**Table 4:** Flow rate scenarios.

Scenarios	Flow rate $(m^3/h)$
1	1100–1250–1400–1300
2	1400–1600–1550–1800
3	1800–2000–1950–2200
4	1100–1800–1400–2200
5	1100–1800–1400–2200
6	1100–1400–1800–2200

The application of the PSO in the parameter’s adjustment of the BELBIC controller for each operating point defined in Table 4, was initially performed empirically by limiting the search space region according to the responses obtained from the controller output, as shown in Table 5. In this way, the search regions will be more restricted, thus, the computational efforts are eased in such a way by that observing the system’s response, the values initially proposed would be evaluated as acceptable or not. Only then, through these simulations, it was possible to obtain the search regions for the solution of the objective function of the optimization problem, where their maximum and minimum limits $(K_1)$ to $(K_5)$, the initial population and velocity functions the implemented equations are presented in Algorithm 1 shown below.

**Table 5:** Upper and lower limits of the parameters of the initial particle function.

Bounds	Alpha	Beta	$K_1$	$K_2$	$K_3$	$K_4$	$K_5$	Description
LB	0.1	0.1	0.001	0.001	0.00001	0.0001	0.0001	Lower bounds of variables
UB	1	1	0.009	0.009	0.00009	0.0009	0.0009	Upper bounds of variables


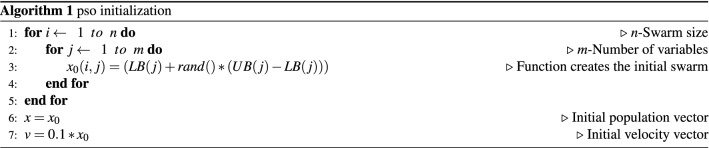


In the BELBIC simulation process using PSO, the updating of particles is done in two steps, given by the Eqs. (19) 3and (20). Where the hyperparameters are described in the Table 6.

**Table 6:** Parameters of Eqs. (19) and (20).

Parameters	Value	Description
*m*	7	Number of variables(Alpha, Beta, $K_1$, $K_2$, $K_3$, $K_4$, $K_5$)
*n*	50	Swarm size
*int*	20	Total number of iterations
$c_1$	2	Cognitive rates^55^
$c_2$	2	Social rates^55^
*w*	0.85	Inertia weight
*rand*	[0,1]	Randomly generated number between 0 and 1
$pbest_{i,j}^{k}$		Best position of particle *i* in dimension *j* at time *k*
$gbest_{j}^{k}$		Best position of the swarm population in dimension *j* at time *k*

To determine the inertial weight *w*, tests were carried out with 20 iterations for different values of *w*, between [0.6 | 1.2]^55^. The best value verified in the tests was 0.85, which presented a better balance with respect to the algorithm’s ability to perform local and global searches, minimizing premature convergence and allowing the particles to accumulate around the global optimum more effectively.

The results of the PSO application in the adjustment of the BELBIC controller parameters are presented in Table 7, showing the output at the end of the simulation process,, was established a restriction of the total number of iterations 20, which is one of the algorithms stopping criteria and the total number of particles 50. Therefore, the average elapsed time for the simulation was approximately 19 minutes.

**Table 7:** PSO Optimization Result.

IAE	Alpha	Beta	$K_1$	$K_2$	$K_3$	$K_4$	$K_5$
7810.504	0.805	0.207	0.0041	0.00216	0.0000527	0.000405	0.000752

As noted, the *bestfun* value represents the value of the IAE, i.e., the minimum value of the objective function. In addition to this value, the values found for the alpha, beta, $K_1$ to $K_5$ parameters are presented, respectively, which is the main objective for using this technique. The algorithm has a quick convergence to the minimum value of the objective function as the number of particles is increased, due to the fact that there is a greater exploration within the solution search spaces, in accordance with the PSO concept; When an individual within a pack encounters a more attractive region (greater number of foods), the rest of the pack is notified; If the other individuals in the pack cannot find a better region, they will all converge upon this most favorable point.

Table 8 presents the remaining scenarios, it can be observed conclusions that the only difference between all the parameters generated through the simulations in Matlab using the PSO is concentrated in the parameter $K_5$, but it is worth pointing out that the values found have numerical values very close because it is an approximation of decimal places.

The simulations for the conditions mentioned in Table 8 were performed using the respective transfer functions without using the PSO, so the results were analyzed according to the behavior of the system, since the PSO had already returned a optimal starting point for the parameters, just fine-tuning the new parameters.

Thus, it appears that this parameter has changed slightly in its value to adapt to the desired behavior of the respective flow rate. For each operating point the parameter values are changed according to the respective scenarios.

**Table 8:** BELBIC Controller Parameters.

Scenario	Alpha	Beta	$K_1$	$K_2$	$K_3$	$K_4$	$K_5$
1	0.8	0.2	0.004	0.002	0.00005	0.0004	0.00075
2	0.8	0.2	0.004	0.002	0.00005	0.0004	0.00060
3	0.8	0.2	0.004	0.002	0.00005	0.0004	0.00050

**Figure 16:**
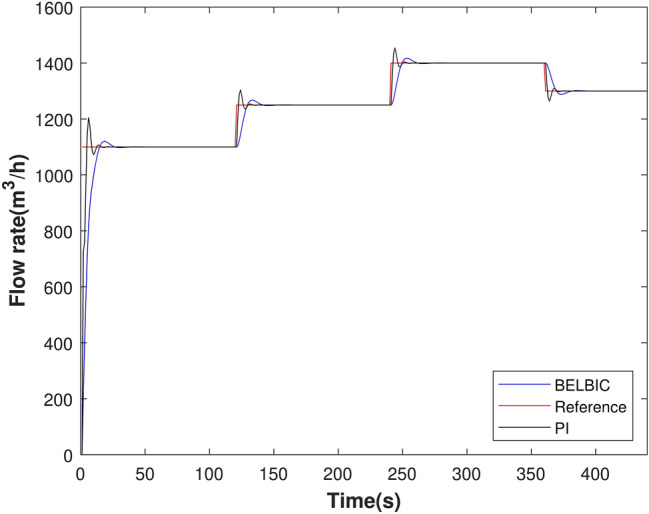
Flow rate control experiment in MATLAB.

The simulation result of BELBIC controller was implemented in MATLAB / Simulink compared to conventional tuned PI. For the analysis of the implemented controllers, a simulation was carried out with scenario 1. Figure 16 shows system simulation response.

Analyzing Fig. 16, It is observed that BELBIC has lower overshoot in all flux reference value transitions, it is also observed that the two controllers eliminate the error in steady-state. A relevant point regarding a water pumping system is that the fact that overshoot occurs during the pumping process will subject the valves and piping to physical wear. The control will provide the pumping system with an increase in hydraulic and energy efficiency, as it will minimize high-pressure peaks in the system and a sudden variation in the acceleration of the electric motors connected to the system, generating an increase in energy consumption.The characteristics of this system are similar to those of our articles^18^ and^56^ and therefore produces results of the same order of magnitude. Articles^19^ and^20^ present systems with other physical characteristics and values of other magnitudes. However, BELBIC also presents better results.

Several techniques can be applied to determine controller parameters. This process is known as controller tuning. Using the method of Åström and Hägglund^59^. However, this method only provides a good starting point and can be used for automatic tuning of simple regulators as well as initialization of more complicated adaptive regulators.

As this method is just a starting point to determine the value of $K_i$ and $K_p$ gains, the first values calculated for these gains were not so good because the response presents a large overshoot. These values were adjusted until the response improved and the values found were $k_p=0.06$ and $k_i=0.045$.

After defining all the respective parameters, shown in Table 8, and with the help of the supervisory screen (Fig. 15) and according to the established scenario, the flow rate steps presented in Table 4 were defined.

Flow rate steps are applied every 2 minutes automatically using a LADDER programming in the CLP, i.e., a change in the flow rate reference value at the given time. By defining all BELBIC controller parameters and their respective steps, the closed-loop tests will be performed and the results obtained in the process using the controller will be presented.

Initially, only for Scenario 1, the signal analysis of the BELBIC controller is performed, justified by the similaties between the remaining scenarios. Therefore, it is necessary to approach the signals generated in the first scenario.

- **Scenario 1**

**Figure 17:**
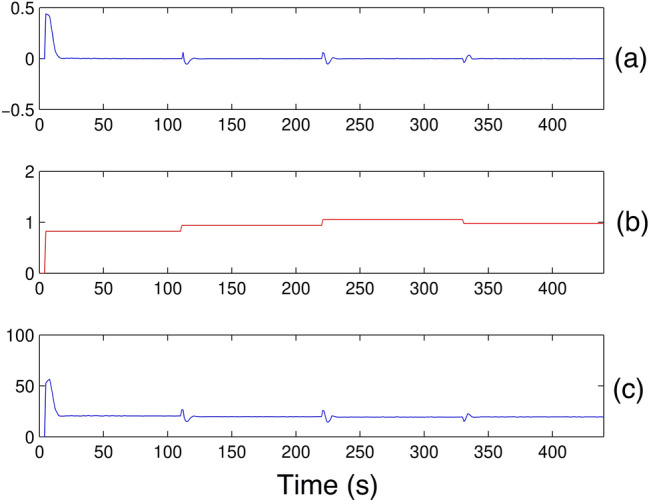
BELBIC Controller Signals: *SI* e *ES*.

As can be seen in Fig. 17a, b (sensorial signals) and Fig. 17c (emotional signal); there is a tracking of the reinforcement (emotional signal), that suffers variation according to the obtained response in Fig. 19.

**Figure 18:**
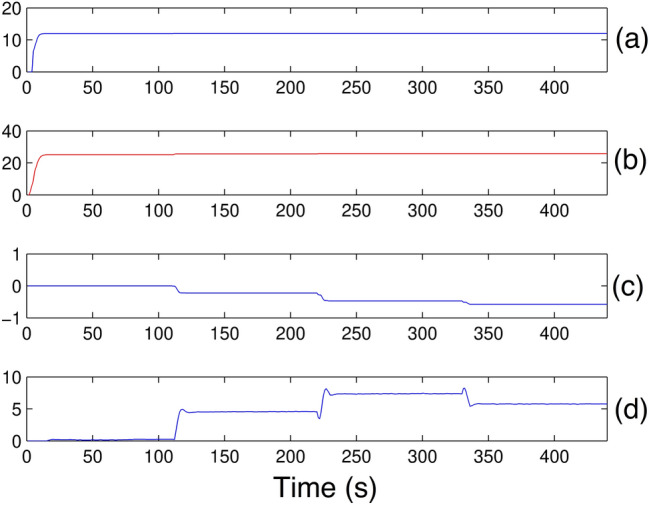
Amygdala and orbitofrontal cortex weights: *V* e *W*.

Changes in sensory and emotional signals do not affect the behavior of the amygdala weight (*V*) in Fig. 18a, b; therefore, the orbitofrontal weight (*W*) Fig. 18c, d makes the necessary inhibition causing the output (*MO*) to have the desired behavior. When the reinforcement reappears, (*W*) may decrease again, allowing the amygdala to express the previously learned association.

In fact, the negative values produced are caused by the high inhibitory effects of the orbitofrontal cortex that not only counteract the excitatory effects of the amygdala, but also produce negative responses whenever the emotional signal is of negative magnitude.

**Figure 19:**
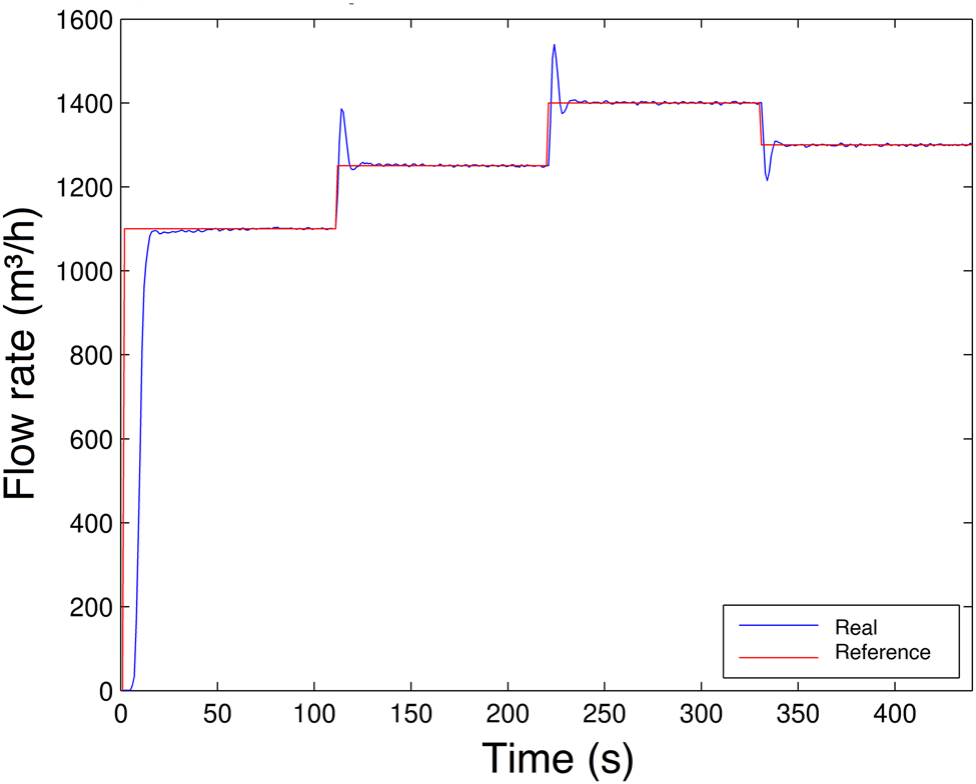
Flow rate control experiment Scenario 1.

To conclude Scenario 1, Fig. 19 shows the controlled plant output acting on a flow rate control. This way, as long as the system flow rate is lower than the reference flow rate, the controller acts by increasing the motor pump rotation speed until the flow rate reaches the reference value. It is observed that the controller practically eliminates the steady-state error and in the transition for the reference value generates an overshoot, which is the highest point that the system transient response reaches. Subsequently, for Scenarios 3, 4, and 5, a solution will be presented to decrease this overshoot.

- **Scenario 2**

Another scenario was defined for the controller analysis. Similar to the first scenario, the parameters and reference values are set through the supervisory screen. The adopted data in this scenario follow in Tables 4 and 8. As seen, the parameters $K_1$ to $K_4$ have the same values as the previous scenario, only differing the parameter $K_5$, even so, with very similar value. The parameter values of this scenario were found using the PSO in a simulation environment.

**Figure 20:**
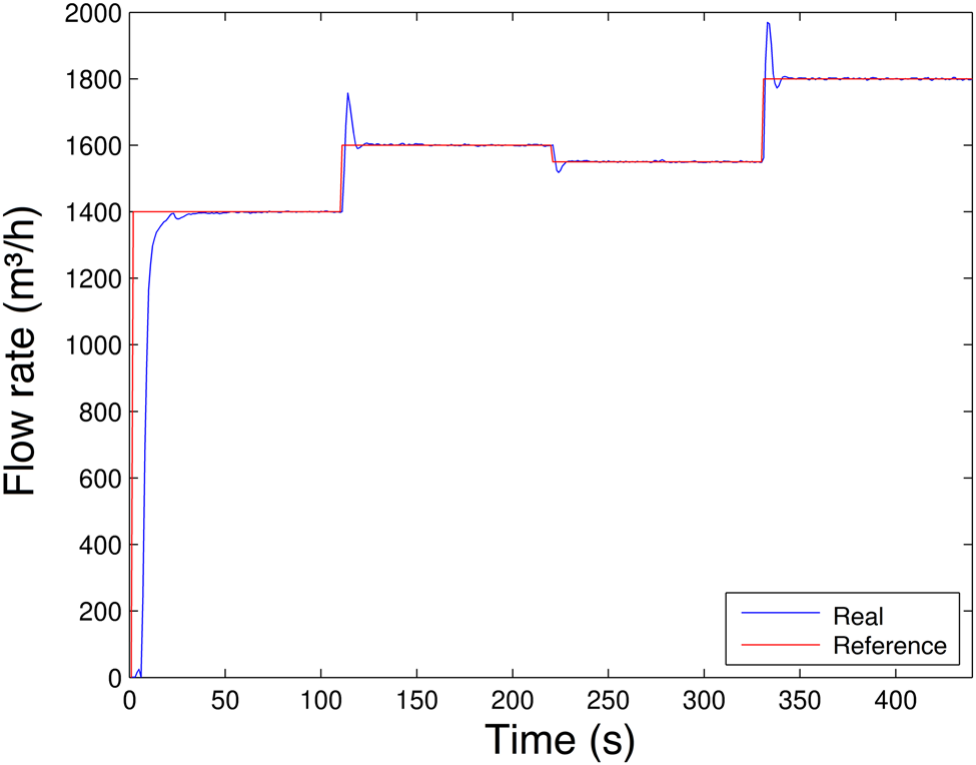
Flow rate control experiment Scenario 2.

The result of Scenario 2 is presented in Fig. 20. As in the first scenario, it is observed that even by varying its flow rate values in other operating ranges, the controller can keep the flow rate with error in steady state almost zero and small overshoot in transient regimes.

- **Scenario 3**

**Figure 21:**
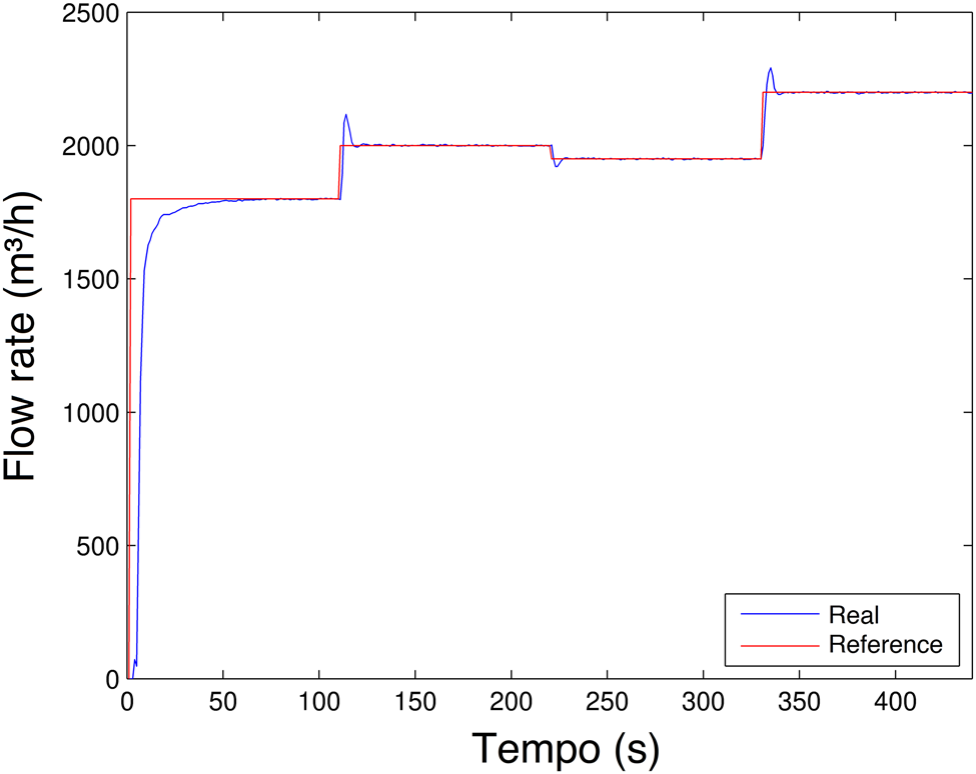
Flow rate control experiment Scenario 3.

Finally, in order to conclude the first three scenarios, the experiment has its result showen in Fig. 21. A transient overshoot (slightly smaller compared to the previous) and a very small steady state error is also observed. To have these steady-state error values as a basis, the experiment data show that the average errors are approximately $\pm 0.8 m^3/h$ and the maximum errors $\pm 5 m^3/h$. The errors found can be considered negligible in relation to the flow rate order of magnitude 2000 $m^3/h$.

Analyzing the three established scenarios, it was observed that in all three cases there was an overshoot signal. In order to ensure that the process output value does not exceed a certain desired value above the applied signal step, which may cause problems or even damage to the system^32^, at first, we decided to use fixed parameters, but in order to compensate the effect of nonlinearities present in the process, we decided to approach multiple models, splitting into operating ranges according to the steps. The idea was to design a system that would change the parameters of the BELBIC controller when the flow rate reference changes in the operating range, so that the controller effectively range and modify the user defined parameters.

As can be seen in Table 9, the parameter $K_5$, of the BELBIC controller, is subdivided into four values empirically based on the values found through PSO optimization$( K_{5,1}, K_{5,2},K_{5,3}~e~K_{5,4})$, which will be changed according to the operating range, i.e., varying the step will change the parameter $K_5$. The result of this technique will be presented below in Scenarios: 5 and 6. We point out that the values obtained for $K_5$, in Table 9, are maximum and minimum values of $K_5$, established in Table 8.

**Table 9:** BELBIC controller parameters for each operating range.

Scenario	$K_{5,1}$	$K_{5,2}$	$K_{5,3}$	$K_{5,4}$
4	0.00075	0.00075	0.00075	0.00075
5	0.00075	0.00047	0.00056	0.00039
6	0.00075	0.00058	0.00048	0.00039

In order to obtain a comparison of what would cause parameter change in the, three new scenarios were defined covering all operating ranges previously established, that is, ranging from 1100 to $2200 m^3/h$. In the first, the parameter $K_5$ remains fixed at 0.00075 for all established ranges of flow rate steps, in order to have a behavior analysis by adopting this possibility. Then, in the second, the values of $K_5$ undergo definite variations based on empirically experiments, but their flow rate step values are the same as in Scenario 5. Finally, the third will also vary $K_5$, only that this time the flow rate steps are increasingly established respecting the same range considered initially. The three conditions taken into account are presented in Table 9.

- **Scenario 4 and 5**

**Figure 22:**
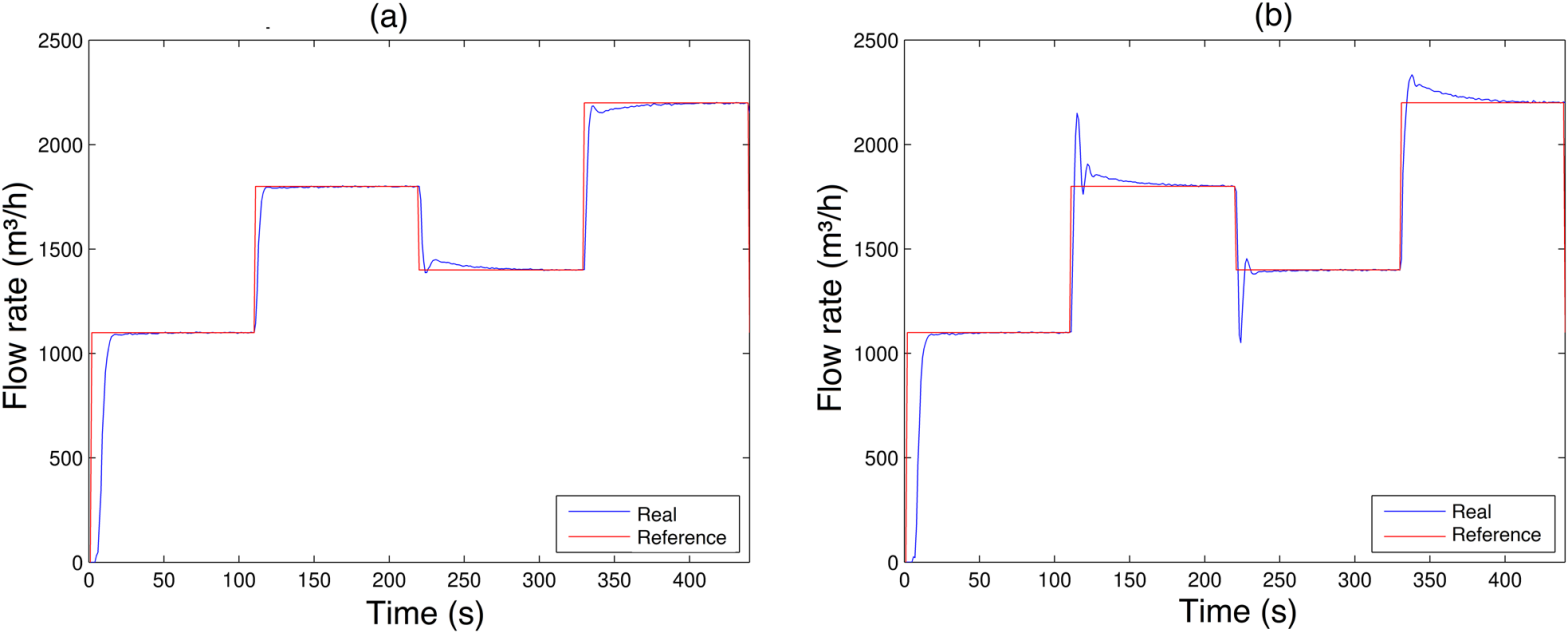
(**a**) Flow control experiment Scenario 4; (**b**) Flow control experiment Scenario 5.

Before applying the parameter change technique, a scenario with a wide range for flow rate variation was constructed, encompassing the values between 1100 to $2200 m^3/h$, so that it would be possible to observe what would happen using the application of fixed parameters, considering the minimum and maximum values established in Table 2. The elaboration of Scenario 4, Fig. 22a, was defined as shown in Table 4, the same as the previous scenarios.

Scenario 5, Fig. 22b, shows the response by applying the parameter change technique, with the same steps as Scenario 4, thus proving the effectiveness of the parameter change.

As observed in Fig. 22a, a transient overshoot occurred and the steady state error is minimized. The result obtained was expected, thus justifying the use of parameter changes to improve the result. Faced with the application in the parameter change $K_5$, as in Table 9, significant improvement was evident, a result quite different from that previously obtained (Scenario 4), which used only a single value for $K_5$. Thus, the system response shown in Fig. 22b shows a significant improvement, clearly observed, the overshoot practically disappeared and the characteristic of eliminating the steady-state error remained. Thus, the system performance presented a satisfactory result, even when the system changes the operating range.

- **Scenario 6**

The last scenario portrays the same, parameter change technique used in Scenario 5. The difference between then is’ that, it was expected to vary the flow reference value within the range of 1100 to $2200 m^3/h$ in a crescent manner.

**Figure 23:**
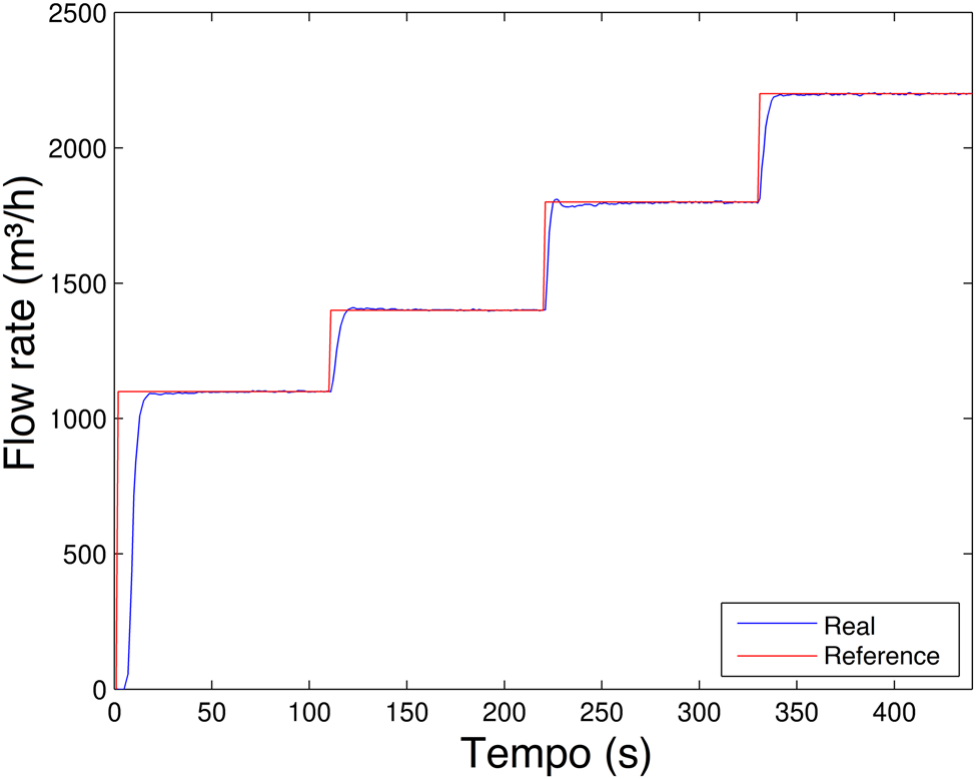
Flow rate control experiment Scenario 6.

The result obtained in Fig. 23 for Scenario 6, as well as for Scenario 5, shows an overshoot and a near zero steady state error, proving once again, high performance of the parameter changing technique. As a result, it was observed that only a simple $K_5$ change would significantly reduce overshoot.

One conclusion after performing all the proposed scenarios is that the experiments that showed changes in $K_5$ indicated that when using this technique, the desired flow rate follows its reference very effectively. Thus, using the parameter changing technique would be a good alternative for other possible scenarios.

Although there is a significant overshoot value in Figs. 19, 20, 21 and 22a; due to the plant’s inertia, the controller presents a rapid stabilization at the setpoint. Considering that there is a greater interest on the part of control systems stability and response time than the overshoot itself, the application of the control is justifiable.

Table 10 presents comparison of the performance of the BELBIC controller using the scenarios proposed.

**Table 10:** BELBIC performance comparison for different scenarios.

Scenarios	Overshoot(%)		RiseTime(Sec)		Settling Time(Sec)	
	Step 1	Step 2	Step 1	Step 2	Step 1	Step 2
1	0.3788	10.8889	4.9463	1.2385	14.8132	108.3398
2	0.3477	9.8567	4.7314	1.2506	18.1522	65.3199
3	0.1543	5.9416	5.5873	1.2303	28.3634	103.0735
4	0	0.1543	5.8873	3.7332	14.9742	7.0057
5	0.0631	19.5367	4.7391	1.6944	15.7378	62.3961
6	0.1893	0.5946	5.4902	5.5386	16.2091	20.4332

In Table 10, it can be seen that the use of the BELBIC in these scenarios, presented different behaviors of dynamic responses, especially in relation to the overshoot, the rise time and the settling time. In this case, scenarios 4 and 6 stand out, obtained from parametric changes for each operating range. Another point to be highlighted was the behavior in all scenarios in the first step, obtaining results of a fast response that justify the presented values of the overshoot and risetime, since the behavior was expected using the BELBIC controller. Furthermore, in subsequent steps the overshoot value has a slight increase that corresponds to the system’s own inertia, however the controller’s response acts quickly to stabilize the system.

The purpose of this paper was to design the BELBIC controller capable of controlling the flow rate of a closed loop pumping system. In the end, the controller would go through a robustness assessment process. The methodology used for this evaluation, varied in operating conditions of the pumping system introduced. These disturbances are random openings through a water recirculation valve located on the main line of the pumping system before the flow meter. Item 7 of Fig. 1 represents the recirculation valve.

The use of this valve will allow to vary its opening in the range of 0–100%, since it is a proportional valve type. For the proposed experiment, the opening was defined in the range of 0% to 40%, i.e., 0% which means no recirculation and 40% implies that there will be only 60% of the water volume passing through the flow rate meter in the main line.

Valve opening values are randomly generated through the supervisory system every 1 minute and then sent to the PLC, which is responsible for opening and closing the valve through the analog output according to the set values.

The idea of creating this condition came from the possibility of randomly simulating potential water consumers on a main line, also found in water supply systems in cities. Thus, the system will have to keep its flow rate constant at a certain reference measurement point no matter, what happens along the way, ensuring the non-interruption for the final consumers.

**Figure 24:**
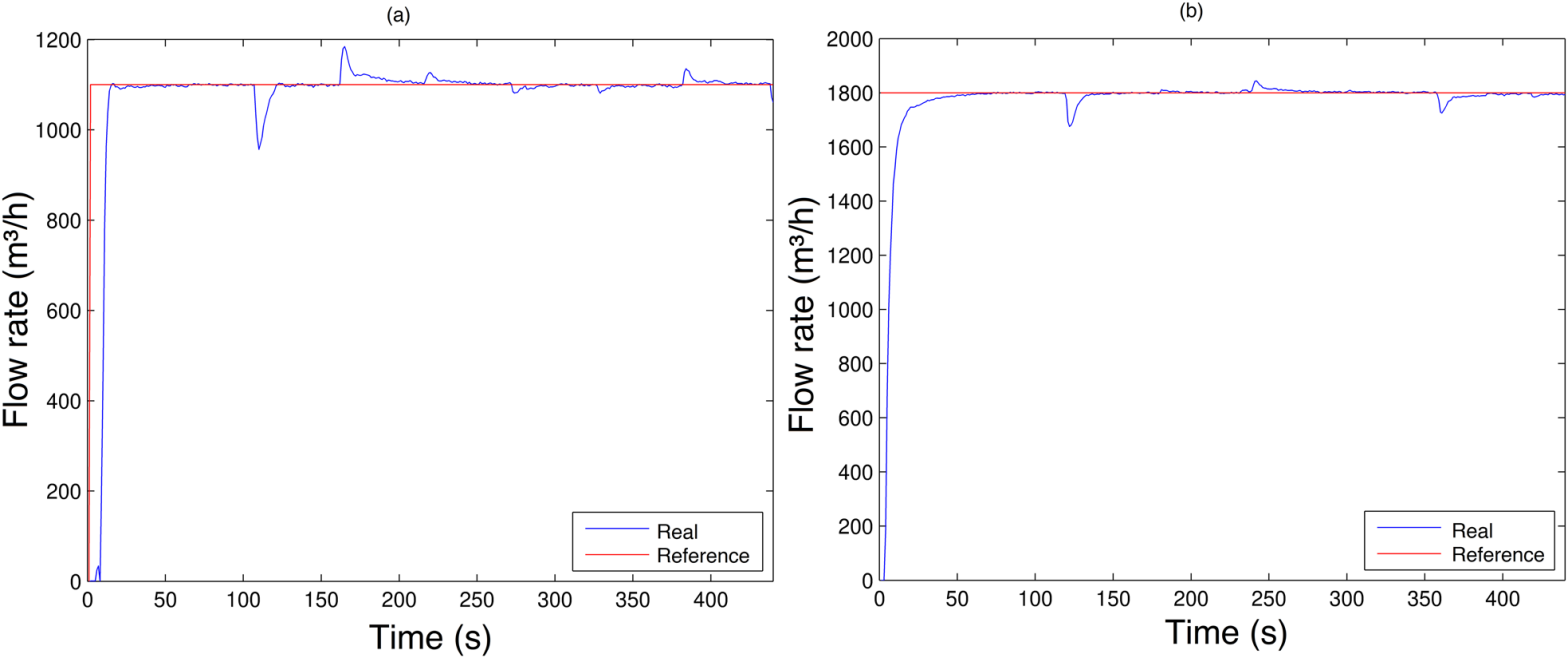
(**a**) Controller robustness assessment (desired flow rate in $1100 m^3/h$); (**b**) Controller robustness assessment (desired flow rate in $1800 m^3/h$).

The results of this proposal to simulate consumers as the controller response, acting on the system to keep the main line flow constant is shown in Fig. 24 for the experiments describe previously.

In the first case, the flow rate was kept at $1100 m^3/h$ and the disturbance was applied. Figure 24a shows that the system was able to compensate, the help of with the controller, stabilizing the system at the desired flow rate. In the second case, Fig. 24b, the flow reference value was $1800 m^3/h$. Similar to the previous case, the result shows the same behavior of the controller acting to maintain the flow rate at the reference value.

Thus, this controller proved to be able to overcome disturbances and keeping the system at the desired value on a permanent basis. Therefore, the results presented were satisfactory even when the process was subjected to disturbances, demonstrating its robustness when applied to nonlinear processes.

## Conclusions

In this article, a new robust BELBIC controller emerges as an alternative capable of achieving satisfactory results for expectations in water pumping systems and industrial processes. This controller has a peculiar characteristic, since it has several parameters that give the freedom to choose the most appropriate set of values for the response^26^. Therefore, this makes it an attractive controller for controling system applications due to its flexibility.

Throughout the article, the crucial objective was to evaluate the control technique based on the emotional learning process of the brain associated with automation, aiming at the implementation of an industrial controller. In particular, we attempted to study and evaluate the characterization of nonlinear systems according to the system operating point; analyze the response of a given system by applying specific stimulus signals, such as the step function. It also presented the limbic system and its computational modeling; to check the available controllers in the industrial environment and finally propose an implementation of a new industrial controller based on the emotional learning process of the brain, applied in a programmable logic controller.

Then, the PSO method was used as an alternative to optimize the controller parameters and minimize the objective function. The application of this method made it possible to find a set of values considered excellent candidates for initial values of these parameters, according to the proposed response, since the authors of BELBIC do not present an alternative for adjustments.

Simulation results, in different operating scenarios, significantly validated a controller which when applied to the proposed system has very satisfactory control performance. Especially in being very efficient in stabilizing the signal and present a fast converging action for the appropriate control signal. This is due to their learning ability. The developed algorithm receives sensory signals and an emotional signal in order to generate the appropriate action in relation to the emotional situation of the system. Therefore, the appropriate choices of the equations governing the emotional and sensory signals of the system, along with the parameters, allows to choose the results according to the output behavior: stabilization time, steady state error and smoothness. Thus, BELBIC becomes an effective and flexible controller with high performance applications. In this context, it makes the BELBIC a potential candidate for industrial implementation.
