# Author Correction: An emotional discrete controller PSO tuned and designed for a real industrial pumping system

**DOI:** 10.1038/s41598-022-10836-2

**Published:** 2022-04-22

**Authors:** Davidson C. Marques, Jeydson L. Silva, Milde Maria S. Lira, Ronaldo R. B. Aquino

**Affiliations:** grid.411227.3 0000 0001 0670 7996 DEE, Federal University of Pernambuco, Recife, 50740-530 Brazil

Correction to: *Scientific reports* 10.1038/s41598-022-08192-2, published online 11 March 2022

The original version of this Article omitted the Funding section. The Funding section now reads:

“This work was supported by Brazilian Coordination for the Improvement of Higher Education Personnel (CAPES) and Federal University of Pernambuco (UFPE) through the PROPG 02/2021 announcement.”

The original Article has been corrected.

